# *In*-*silico* screening of *Schistosoma mansoni* Sirtuin1 inhibitors for prioritization of drug candidates

**DOI:** 10.1186/s40064-016-1891-4

**Published:** 2016-03-07

**Authors:** Raghvendra Singh, Birendra Singh Yadav, Swati Singh, Paras Nath Pandey, Ashutosh Mani

**Affiliations:** Institute of Interdisciplinary Studies, Center of Bioinformatics, Nehru Science Center, University of Allahabad, Allahabad, 211002 India; Department of Biotechnology, Motilal Nehru National Institute of Technology Allahabad, Allahabad, 211004 India; Department of Mathematics, University of Allahabad, Allahabad, 211002 India

**Keywords:** Intestinal schistosomiasis, Sirtuin 1, Sirtinol 1, Molecular docking, ADMET profiling

## Abstract

Schistosomiasis is a common, neglected parasitic disease caused by *Schistosoma mansoni.* Availability of two specific drug oxamniquine and praziquintel for treatment of the disease is a major concern. Recently NAD+ dependent lysine deacetylases have been identified as new drug targets in pathogens. Sirtuins are NAD+ dependent lysine deacetylases that are involved in a wide variety of vital cellular processes. Amongst them, members of sirtuin’s class1 proteins are considered to be main target of the drugs. Sirtinol and Salermide are two known inhibitors of *Schistosoma mansoni* Class1sirtuin which is a protein with unknown 3-D structure. Here, we investigate molecular insights of interaction between modeled sirtuin1 structure and it’s inhibitors, that were derivatives of Sirtinol and Salermide, to prioritize them for their binding affinities with target. A detailed examination of absorption, distribution, metabolism and toxicity of these inhibitors has also been included in the study. Finally we found two derivatives of Sirtinol to be most appropriate drug candidates for Schistosomiasis.

**Electronic supplementary material:**

The online version of this article (doi:10.1186/s40064-016-1891-4) contains supplementary material, which is available to authorized users.

## Background

Schistosomiasis, one of the most common parasitic diseases in developing countries, is caused by species of dioecious blood flukes belonging to the genus Schistosoma. After malaria, schistosomiasis is the most important tropical disease in terms of human morbidity. Under Schistosomiasis control initiative more than 40 million doses of Praziquantel have been dispensed in sub-Saharan Africa (Fenwick et al. 2009). Praziquantel is reported as the only drug used for mass treatment of schistosomiasis (Dömling and Khoury 2010) while Oxamniquine is a specific drug for *Schistosomiasis mansoni* (Cioli 1993; Fallon 1994). So, availability of the limited drug for the disease draws attention towards the search for new therapeutic targets as well as development of novel compounds to overcome the prospective threats from resistant strains of schistosomes (Doenhoff et al. 2008) that have been already reported and characterized in endemic areas (Melman et al. 2009).

Recently NAD+ dependent lysine deacetylases (Histone modifying enzymes) have been identified as new drug targets in several pathogen (J Pierce et al. 2012). Sirtuin1 protein in *Schistosoma mansoni,* a member of NAD+ dependent deacetylases family which is phylogenetically unrelated to the Zn^2+^-dependent deacetylase (Frye 2000), has been targeted in assays designed to study the therapeutic effect of inhibitors (Lancelot et al. 2013). Sirtuin proteins have been classified into five different classes (I, II, III, IV and U), on the basis of presence of conserved motifs in their core domain (Religa and Waters 2012). Parasitic class I sirtuins, characterized by the GAGXSXXXGIPDFRS, PS/TXXH, TQNID and HG motifs (Religa and Waters 2012) have been extensively and successfully explored as antiparasitic targets (Vergnes et al. 2002). It has been reported that these proteins have vital role in parasite survival by catalyzing the deacetylation reaction of acetylated lysine residues of nuclear histones and other substrates, with NAD+ as a cofactor (Vergnes et al. 2002). Salermide, which induces cell death in *S. mansoni* by targetting both Sirt1 and Sirt2 (Lara et al. 2009), is a potential anticancer agent due to it’s sirtuin inhibition property. The inhibition of sirtuins has been less explored for their therapeutic use against parasites. The molecular features of SmSirt2 as well as it use for the development of new targets for schistosomiasis were explored in a recent studies (Singh et al. 2015; Singh and Pandey 2015). In the present paper Sirt1 protein of *S. mansoni* has been used for the study. Due to unavailability of determined three dimensional structure of *S. mansoni* Sirt1 protein molecular insights of the inhibitor protein interaction or their participating residues are not known. Here we have modeled a 3-D structure of the protein by multi-template homology modeling. After that ten derivatives of salermide and sirtinol were screened against the modeled structure by docking. For sorting the inhibitors according to their druggability they were assessed on ADMET parameters.

## Methods

### Sequence retrieval and phylogenetic analysis

Sirt1 protein sequence of *S. mansoni* was obtained from Uniprot (Acession no. A6XDL2). Physicochemical properties were predicted by using ProtParam server (http://web.expasy.org/protparam/). BLASTp (Altschul et al. 1990) program was used to search similar protein sequences against non-redundant protein database in NCBI. The Sirt1 amino acid sequence was used as query sequence and identical amino acid sequences present in different species were selected for further study **(**Table 1). The Multiple Sequence Alignment of protein sequences was performed using ClustalW 2.0.10 program (Larkin et al. 2007). MEGA5.2 (Tamura et al. 2011) was used for constructing and analysing the phylogenetic tree. The neighbor-joining method (Saitou and Nei 1987) was used to get the information of evolutionary history. All the characters were having equal probability for transition. The 10,000 replicates of bootstrap consensus were taken to represent the evolutionary history of the taxa (Felsenstein 1985). Branches having less than 50 % bootstrap replicates were sorted out. The percentage of replicate trees in which the associated taxa clustered together in the bootstrap test (1000 replicates) is shown next to the branches. The tree is drawn to scale with branch lengths in the same units as those of the evolutionary distances used to infer the phylogenetic tree. The evolutionary distances were computed using the Poisson correction method and are in the units of the number of amino acid substitutions per site. All positions containing gaps and missing data were eliminated from the dataset (complete deletion option). BioEdit 7.0.2 (Hall 1999) has been used to calculate the entropy.

**Table 1:** Comparison of DOPE score, quality factor determination through ERRAT and stereochemical property generated by Ramachandran plot of five models predicted through MODELLER

	Residues in most favored regions	Residues in additional allowed region	Residues in generously allowed region	Residue in disallowed region	DOPE Score	Overall quality score errat
Model1	0.858	0.125	0.009	0.009	−43,519.9	53.971
*Model2*	*0.906*	*0.083*	*0.009*	*0.002*	*−44,363.7*	*60.000*
Model3	0.875	0.112	0.009	0.004	−43,488.5	48.381
Model4	0.877	0.116	0.004	0.002	−43,591.1	56.883
Model5	0.891	0.096	0.011	0.002	−43,627	55.052

### Multi-template homology modeling of Sirt1 protein

PSI-BLAST (Altschul et al. 1997) algorithm was used against the Protein Data Bank (www.pdb.org) to search homologous sequences having 3D structure solved by experimental techniques. After PSI-BLAST search 50 protein structures were found then after three structures having PDB ID 2hjh, 4i5i and 4iao matching with different positions of query sequence were used as a template. Multi-template modeling was performed by MODELLER 9.10 (Šali and Blundell 1993). Homology modeling of was done by the steps: template selection from psi-blast, sequence multiple template alignment, multiple model building, evaluation of model, model refinement and model validation (Martí-Renom et al. 2000).

### Protein structure optimization, quality assessment and visualization

MODELLER generated several preliminary models which were ranked based on their DOPE and GA scores. Models with low DOPE score were selected and stereo chemical property of each model was checked by PROCHECK. The model which having low DOPE score and least number of residues falling in disallowed region in Ramachandran plot was selected for further study. ProSA-Web server (Wiederstein and Sippl 2007) was used to check the quality of models, energy and stereochemical geometry.

### Active sites prediction and ligand designing

Active site in the protein model were identified by using DoGSite sever (Volkamer et al. 2012). Scoring was based on a linear combination of the three descriptors volume, enclosure and hydrophobicity for all the pockets present in the protein.

Sirtinol and Salermide are two known lead compounds against the Sirtuin Protein. Four derivatives of salermide were generated by using Zinc database on the basis of similarity. The four derivatives of sirtinol were constructed by Chemsketch11.0.[ACD/Structure Elucidator, version 11.0, Advanced Chemistry Development, Inc., Toronto, ON, Canada, www.acdlabs.com, 2008].

### Molecular docking

Pre-docking steps were performed with the help of AutoDockTools 4.2 in which grid was generated near the active site residue Ala103 (GlyAlaGly domain) (Morris et al. 2009). Molecular docking was performed by Autodock 4.2. Flexible docking algorithm was used to check the interaction between ligand and protein. Average Grid points used in this docking were (60, 60, 60) and centre of ligand was approximately as coordinates: [−3.879 36.765 93.619]. Grid point spacing was 0.375Å. Lamarckian genetic algorithm (LGA) was used for docking.

### ADMET screening

In order to rank the putative drug candidates on the basis of Absorption, Distribution, Metabolism, Excretion and Toxicity (ADMET) properties the structures of salermide, sirtniol and eight derivatives were done by using admetSAR (Cheng et al. 2012). First the structures of ligand PDB files were converted to SMILEs files by using Online SMILES Translator and Structure File Generator of National cancer Institute (http://www.cactus.nci.nih.gov) and these files were used as input for ADMET prediction.

## Results and discussion

### Physicochemical properties

The *S. mansoni*. Sirt1 protein is 568 amino acids long and was predicted to have a molecular weight of 63,262.3 Daltons with an isoelectric point (pI) of 4.71. Sirt 1 has a Negative GRAVY index of −0.513 which is indicative of a hydrophilic and soluble protein.

### Homology modeling and structure validation

The selected template protein’s PDB IDs are 2hjh, 4i5i and 4iao. These template proteins showing 40, 46 and 46 % identity with target protein sequence, respectively. The multiple template modeling was done by using these three protein structures.

**Fig. 1:**
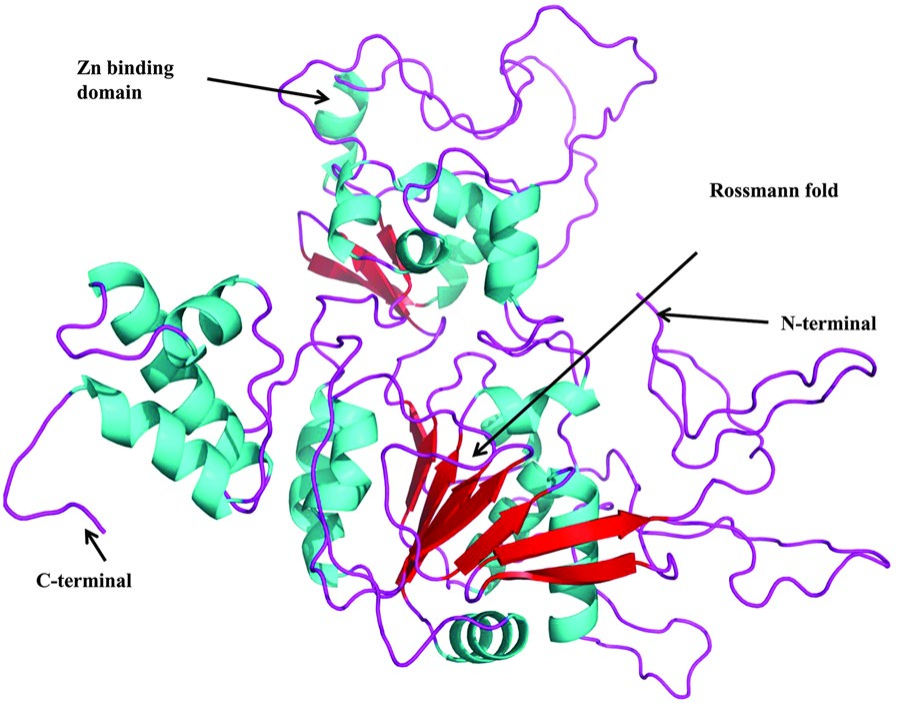
3-D model of sirtuin1 protein of *Schistosoma mansoni* predicted by multi-template modeling

For selection and validating the modeled structure (Fig. 1) obtained from the Modeller 9.13, DOPE score and Ramachandran plot was drawn using PROCHECK (Ramachandran plot has been shown in Additional file 1: Figure S1), quality score generated by ERRAT server were used. On the basis of DOPE score value (Table 1) model2 was selected for the further downstream analysis. Selected model subjected for validation step by`analyzing steriochemical property and quality score generated by ERRAT server. Steriochemical property and ERNET score of all the modeled structures are compared (Table 1). Ramachandran plot of Selected model are shown in Additional file 1: Figure S1 have the phi/psi angles of 90.6 % residues are in the most favored region, 8.3 % residues are in additional allowed region, 0.9 % residues are in generously allowed region and 0.2 % residues are in disallowed region (Additional file 1: Figure S1). Analysis suggested that model2 is best representative of Sirt1 with respect to Ramachandran plot and ERNET score and further selected for downstream analysis.

The overall protein quality and its structural deviation from the total energy were measured by Z-Score (Additional file 1: Figure S2). The black point in Additional file 1: Figure S2 represents the Z-score of the protein. Groups of structures determined from different source (NMR, X-ray) are distinguished by different color (NMR with dark blue and X-ray by light blue color). The plot of Z-Score represents value of the modeled protein of *Schistosoma mansoni* is (4.5) is located within the space of proteins related to NMR. The modeled protein’s Z-Score is within the acceptable range (−10 to 10, negative Z-score are good and depend on length of protein).

Modeled protein has large number of insertion in both the terminals (Fig. 1). Explicitly blast tool was used to find sequence similarity of inserted regions but it did not give any kind of similarity with existing annotated sequences.

### Active site prediction

The probable binding sites and other sub binding site were detected in the predicted model of sirt1 and analyzed in terms of their geometrical, as well as physico-chemical properties. The DoGSite Scorer predicts eighteen binding pockets in terms of calculated descriptors as shown in Table 2. The ligand generally forms favorable interactions with the binding sites having largest interacting cavity, so active pocket P0 can be predicted as potential binding pocket. Amino-acid composition descriptors calculate the values in term of non-polar amino acid ratio, polar amino acid ratio, positively charged amino acid ratio and negatively charged amino acid ratio and they are 0.45, 0.34, 0.22 and 0.09 respectively. The most favorable binding site contains amino acids with high conserved residue score and amino acid residues: Ala, Arg, Asn, Asp, Cys, Gln, Glu, Gly, His, Ile, Leu, Lys, Met, Phe, Pro, Ser, Thr, Trp, Tyr and Val are responsible for constituting potential active-site of situin1 structure.

**Table 2:** Pockets and descriptors calculation for Sirtuin1 model

Name	Volume [Å]^3^	Surface [Å]^2^	Lipo surface [Å]^2^	Depth [Å]	Simple score
P0	2117.23	2817.48	1667.61	27.66	0.6
P1	665.35	852.27	558.99	18.26	0.44
P2	605.27	765.08	445.91	16.5	0.33
P3	482.42	960.84	633.42	12.77	0.32
P4	442.11	793.97	462.03	16.59	0.24
P5	281	618.08	400.35	13.89	0.15
P6	241.21	180.08	175.7	18.94	0
P7	213.87	330.72	200.03	8.92	0.04
P8	196.15	322.3	206.77	10.56	0
P9	186.27	436.36	274.88	10.81	0
P10	183.57	233.86	187.31	10.71	0
P11	167.65	409.83	251.77	9.69	0.02
P12	144.29	285.9	169.88	7.47	0
P13	138	416.77	291.98	9.49	0
P14	135.17	336.34	263.55	9.61	0
P15	130.3	359.5	268.5	8.44	0
P16	122.72	361.74	153.14	6.71	0
P17	115.41	179.24	149.15	10.5	0
P18	112.84	247.52	203.21	9.15	0.05
P19	112.07	299.23	219.08	7.94	0
P20	111.81	343.81	194.84	10.21	0
P21	105.14	200.69	145.01	6.16	0
P22	103.34	112.15	76.41	11.79	0

### Molecular docking

**Fig. 2:**
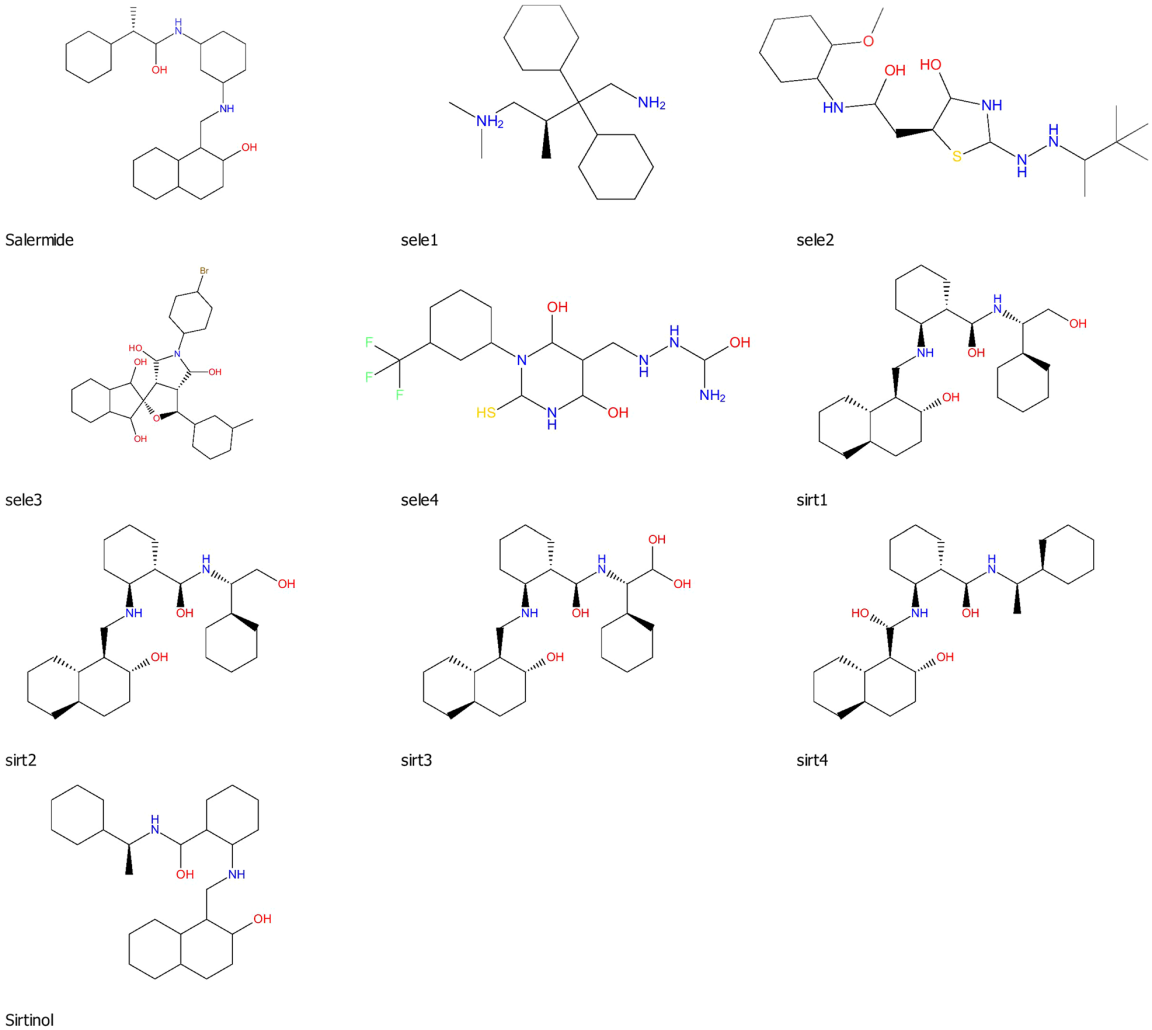
Selected ligands and their analogs used of molecular docking with hSirt1 and SmSirt1. *Sale1–4* represents the analogs of salermide and *sirt1–4* represents derivatives of sirtinol

For the molecular docking, reported inhibitors of class I sirtuin known as sirtinol and salermide were selected. In this study we have generated four derivatives each of sirtinol and salermide. The structures of ten inhibitors have been shown in Fig. 2. These selected ligands further used for for comparative docking with Sm SirtI and Hs SirtI. The binding affinity of sirtinol and salermide is −10.36 and −11.25 kcal/mol with respect to Sm sirtuin1 respectively. For *Homo sapiens* Sirtuin1 the binding affinity of sirtinol and salermide is −10.77 and −9.94 kcal/mol respectively. The sirtinol inhibitor shows slightly higher binding affinity towards Hs Sirt1 with respect to Sm Sirt1. Data of docking results has been summarized in Table 3. 2-D plots of the interaction of ten inhibitors with Human Sirt 1 has been shown in Additional file 1: Figure S3, and the 2-D plots for interaction of ten inhibitors with Schistosoma Sirt1 has been shown in Additional file 1: Figure S4.

**Table 3:** Docking results of Human and pathogen Sirtinol1 proteins with 10 drug candidates

Ligand	Binding affinity with host Sirt1 (kcal/mol)	Binding affinity with pathogen sirt1 (kcal/mol)	Pathogen Sirt1		Host Sirt1	
			Ligand efficiency	IC(nM)	Ligand efficiency	IC(nM)
Sirtinol	−10.77	−10.36	−0.35	25.57	−0.36	12.78
Sirtinol1	−9.25	−11.01	−0.36	8.5	−0.3	165.4
Sirtinol2	−5.44	−11	−0.35	8.95	−0.18	0.10333
Sirtinol3	−5.53	−9.2	−0.29	180	−0.17	0.08797
Sirtinol4	−10.51	−10.33	−0.33	26.95	−0.34	19.9
Salermide	−9.94	−11.25	−0.36	5.62	−9.94	52.09
Salermide1	−7.53	−8.34	−0.4	769.44	−7.53	0.00304
Salermide2	−8.43	−5.31	−0.2	127.58	−8.43	666.91
Salermide3	−8.21	−6.35	−0.19	22.22	−8.21	960.99
Salermide4	−8.57	−9.48	−0.36	112.13	−8.57	519.85

### ADMET screening

Druggability and toxicity of ten drug candidates were assessed on the basis of 23 ADMET parameters. The results of ADMET screening have been shown in Additional file 1: Table S1.

### Phylogenetic analysis

**Fig. 3:**
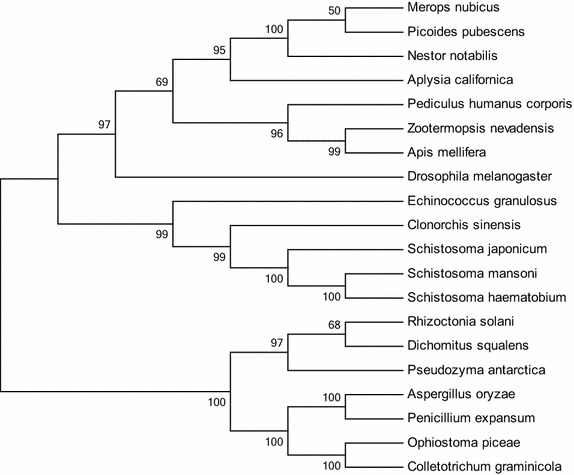
Phylogenetic analysis of sirtuin1 protein homologues from 20 species was constructed by the neighbor-joining method using the MEGA 5.2 program. *Bootstrap values* are indicated against each branch. Phylogenetic analysis showed two large clusters of ESX homeobox-1 protein

The Neighbor-Joining method was used to construct the phylogenetic tree. The minimum branch length lies between *S. mansoni* and *Schistosoma haematobium, Schistosoma japonicum, Clonorchis sinensis* 0.00, 0.06 and 0.25 respectively. In Fig. 3 the phylogenetic tree with bootstrap value are shown, that is approximately 100 for similar sequences. The percentage of replicate trees in which the associated taxa clustered together in the bootstrap test (10,000 replicates) is shown next to the branches. The p distance model was used to construct the evolutionary distances and branch length represents the number of expected amino acid substitutions per site. On the basis of evolutionary distance of 20 sequences, as mentioned in Table 4, related with sirt1 protein phylogenetic tree was constructed and two major clades were obtained and bootstrap values were also calculated through neighbor-joining method.

**Table 4:** Sirtuin1 protein of *S. mansoni* sequence and its different homologues of species with their length and NCBI accession code

S. No.	Organisms	Acession no.	Length
1	*Schistosoma mansoni*	KGB39559.1	568
2	*Schistosoma japonicum*	CAX8242.1	410
3	*Schistosoma haemotobium*	KGB39559.1	683
4	*Clonorhis sinensis*	GAA56043.1	600
5	*Echinococcus granulosum*	CDS22978.1	740
6	*Zootermopsis nevadensis*	KDR10345.1	882
7	*Pediculas humans coporsis*	XP_002432110.1	590
8	*Drosophila melangoaster*	AA047879.1	483
9	*Rhizoctonia solani*	EUC5131.3	535
10	*Aspergillus oryzae*	XP_001820107.2	493
11	*Merops nubicus*	KFQ33949.1	602
12	*C. higginsianum*	EFQ33543.1	532
13	*Pencillium expansum*	KGO37944.1	486
14	*Nestor notabillis*	KFQ50963.1	602
15	*Ophiostoma piceae*	EPE06021.1	631
16	*Pseudozyma antarctica*	GAK62857.1	584
17	*Dichomitus squalens*	XP_007362890.1	581
18	*Picoides pubescens*	KFV72792.1	601
19	*Aplysia californica*	XP_005095144.1	922
20	*Apis mellifera*	XP_006569399.1	865

## Discussion

**Fig. 4:**
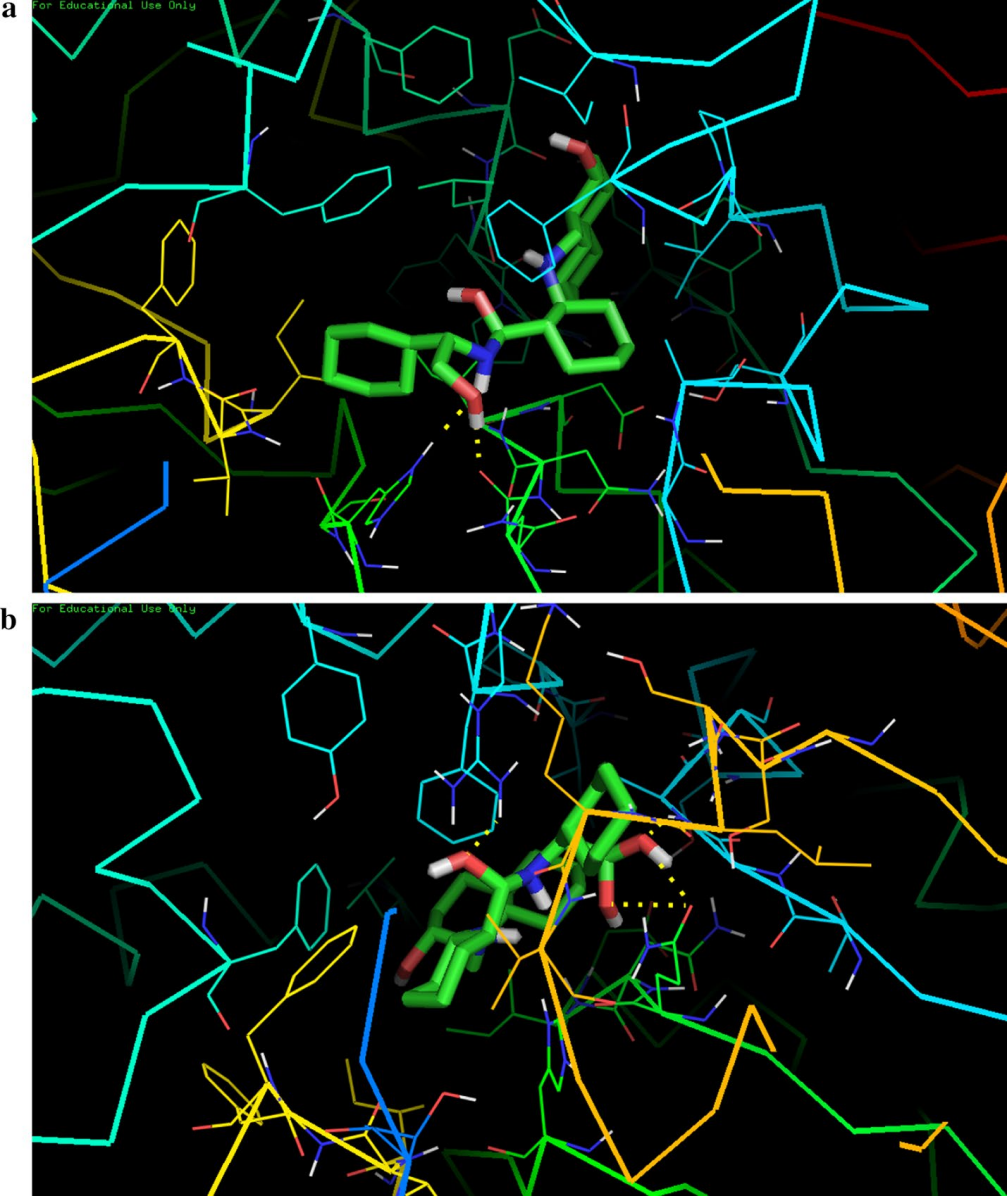
**a** Interaction between sirt2 inhibitor and pathogen sirtuin1 protein. **b** Interaction between sirt3 inhibitor and pathogen sirtuin1 protein

Previous studied by Lancelot et al. depicts that transcriptional knockdown of SmSirt1by RNA interference in *Schistosoma* led to morphological changes in the ovaries characterized by a marked increase in mature oocytes, reiterating the effect of sirtuin inhibitors and suggesting that SmSirt1 may act as principal drug target. The stability of different drug-receptor complexes on the basis of their docking based binding affinities suggests that salermide and sirtinol both have limited preferential binding affinities for pathogen sirt1 protein in comparison to host homolog. Docking based screening suggests that sirtinol2 and sirtinol3 have considerably higher binding affinity with the parasite receptor (Fig. 4a, b). Sirtinol 1, salermide, salermide1 and salermide4 are having slightly higher binding affinities with pathogen Sirt1 protein.

On the other hand the ADMET screening data suggest that sirtinol1 and salermide due to having AMES toxicity may not be considered as drug candidate. Salermide1 is a predicted carcinogen in the AMES tat and salermide4 is a P-glycoprotein inhibitor. Sirtinol3 has been predicted as a drug candidate which has neither blood–brain barrier crossing nor human intestine absorption capability. So, this may have prospects of being used as injection drugs that are directly mixed in blood. Further sirtinol2 is also a promising drug candidate for treatment of Schistosmiasis. Though sirtinol2 is not having blood brain carrier permeability but it is not a major issue as the location of the pathogen in host is intestine.

## Conclusions

Aim of this study was to design and screen new drugs for treatment of Schistosomiasis which is a disease prevalent in tropical regions of the world. We have screened two drug candidates named as sirtinol2 and sirtinol3, both are derived for the structures of sirtinol, a reported inhibitor of *Schistosoma mansoni.* There is need to search the druggability of the screened drug candidates which may prove to be promising in the way of providing low cost and safe drugs to the needy population in the society. This study will trigger the drug development and prospective clinical trial process for schistosomiasis may prove to be a milestone to new drug discovery.

## Additional file

10.1186/s40064-016-1891-4 **Figure S1.** Ramachandran plot of modeled Sirt1. **Figure S2.** Z-score plot shows reliability of modeled structure. The black dot represents Z-score of modeled Sirtuin1 protein of *S. mansoni*. **Figure S3.** 2D plots of all the inhibitors human Sirt1. Here 1 represents salermide, 2 with sirtinol, 3, 4, 5 and 6 with sale1, sale2, sale3, sale4 and sale5 and 7, 8, 9 and 10 with sirt1, sirt2, sirt3 and sirt4. **Figure S4.** 2D plots of all the inhibitors with Schistosoma Sirt1. Here 1 represents salermide, 2 with sirtinol, 3, 4, 5 and 6 with sale1, sale2, sale3, sale4 and sale5 and 7, 8, 9 and 10 with sirt1, sirt2, sirt3 and sirt4. **Table S1.** ADMET screening results.
