# Supplementary material for: In-silico screening of Schistosoma mansoni Sirtuin1 inhibitors for prioritization of drug candidates
Source: Springerplus. 2016 Mar 7;5:286. doi: 10.1186/s40064-016-1891-4 (PMC4781818; doi:10.1186/s40064-016-1891-4)
Supplement: Supplementary file 1 — 10.1186/s40064-016-1891-4 Figure S1. Ramachandran plot of modeled Sirt1. Figure S2. Z-score plot shows reliability of modeled structure. The black dot represents Z-score of modeled Sirtuin1 protein of S. mansoni. Figure S3. 2D plots of all the inhibitors human Sirt1. Here 1 represents salermide, 2 with sirtinol, 3, 4, 5 and 6 with sale1, sale2, sale3, sale4 and sale5 and 7, 8, 9 and 10 with sirt1, sirt2, sirt3 and sirt4. Figure S4. 2D plots of all the inhibitors with Schistosoma Sirt1. Here 1 represents salermide, 2 with sirtinol, 3, 4, 5 and 6 with sale1, sale2, sale3, sale4 and sale5 and 7, 8, 9 and 10 with sirt1, sirt2, sirt3 and sirt4. Table S1. ADMET screening results. [file 40064_2016_1891_MOESM1_ESM.docx]

**Supplementary Information:**

**
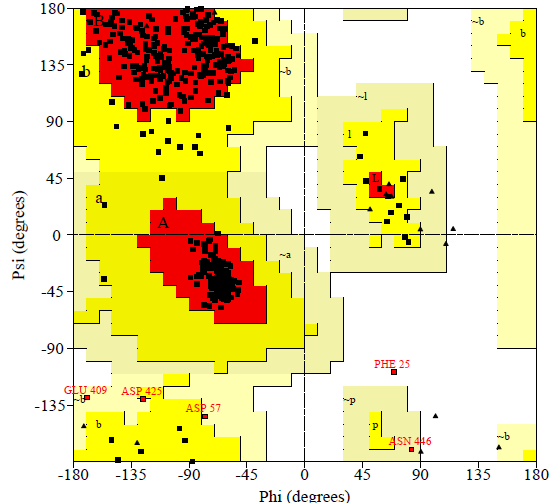
**

**Figure S1.** Ramachandran plot of modeled Sirt1.


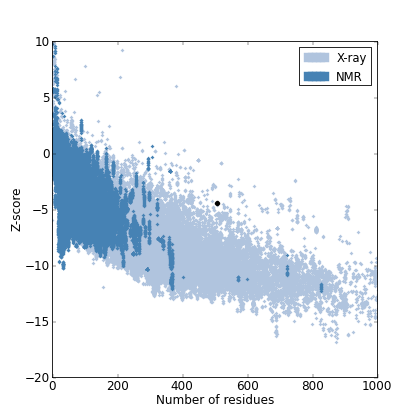


**Figure S2.** Z-score plot shows reliability of modeled structure. The black dot represents Z-score of modeled Sirtuin1 protein of *S. mansoni*


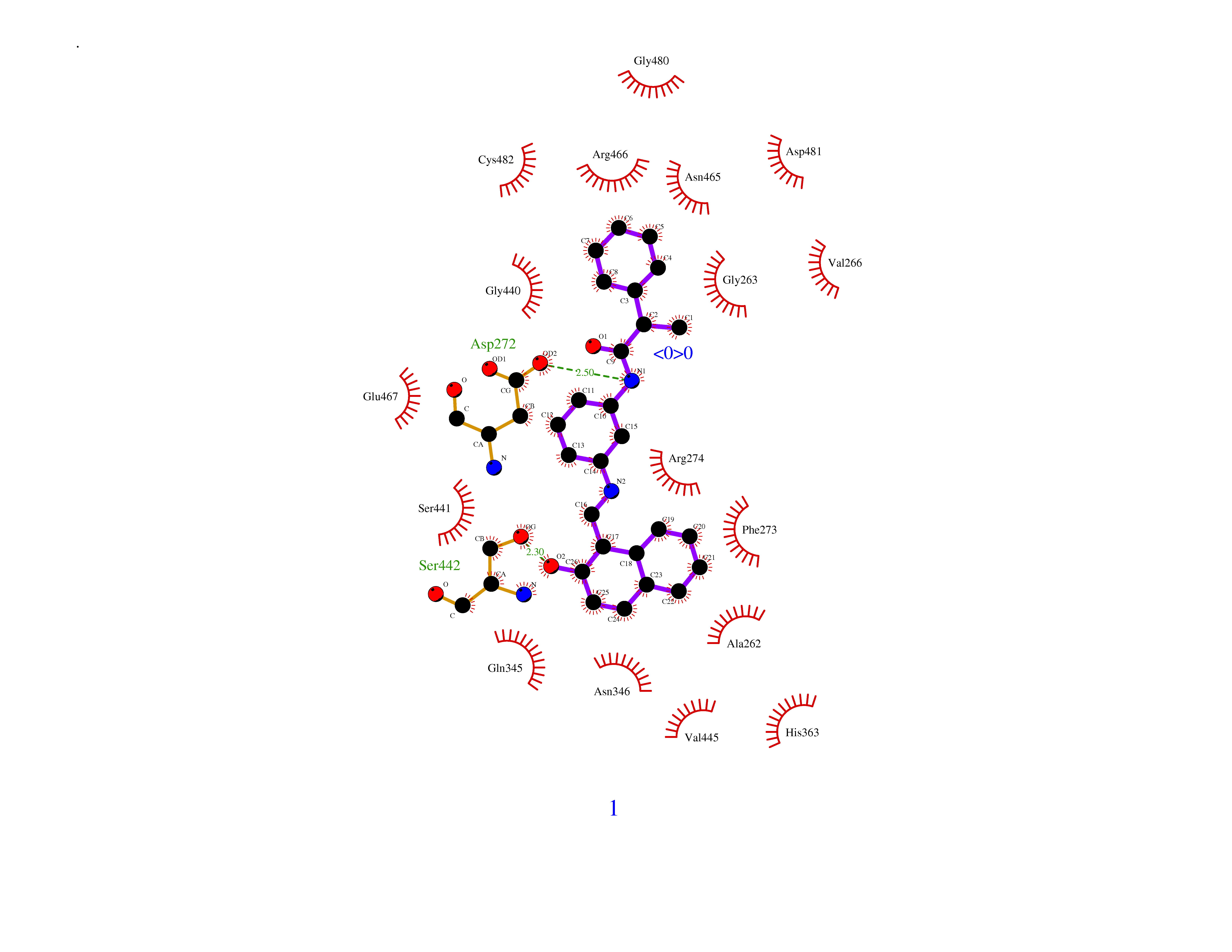

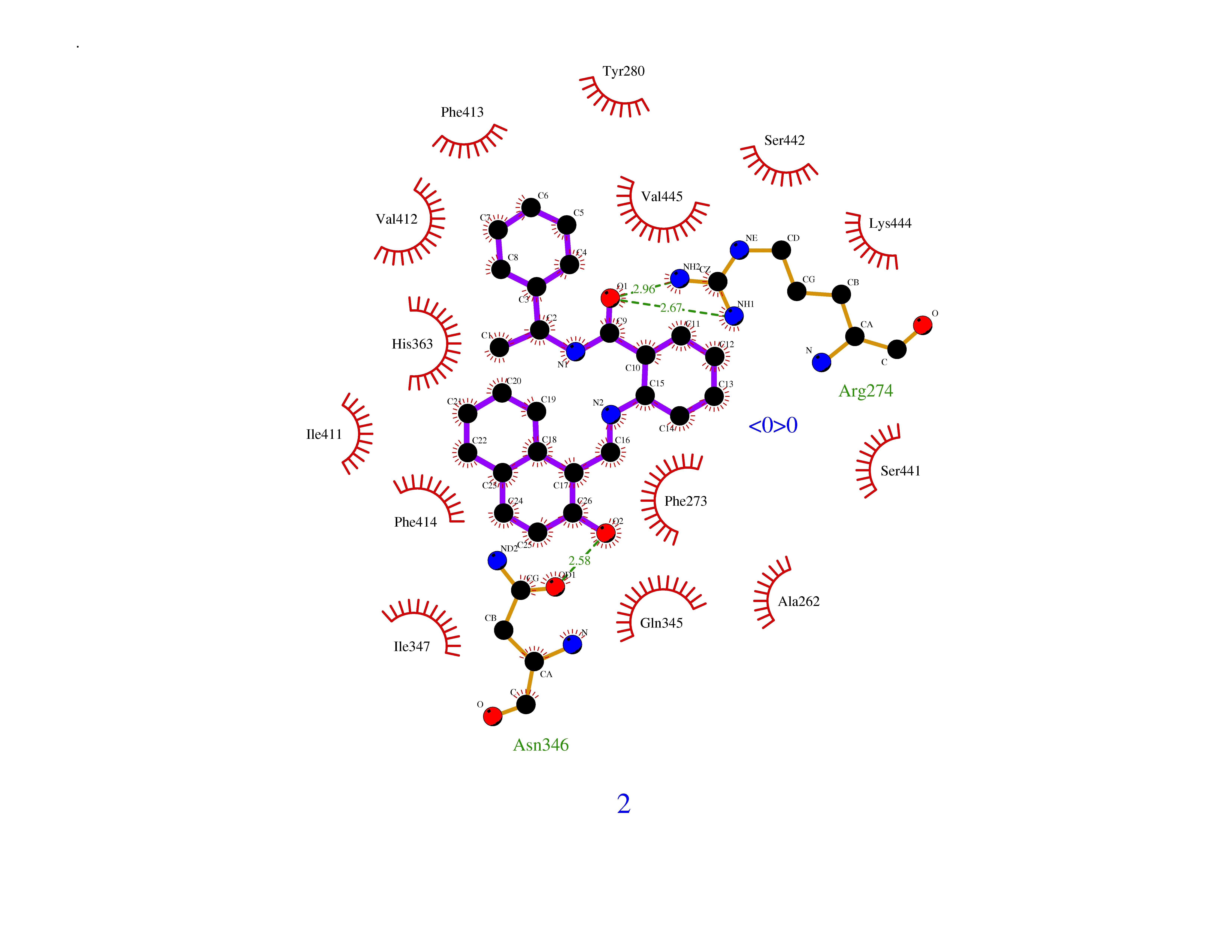

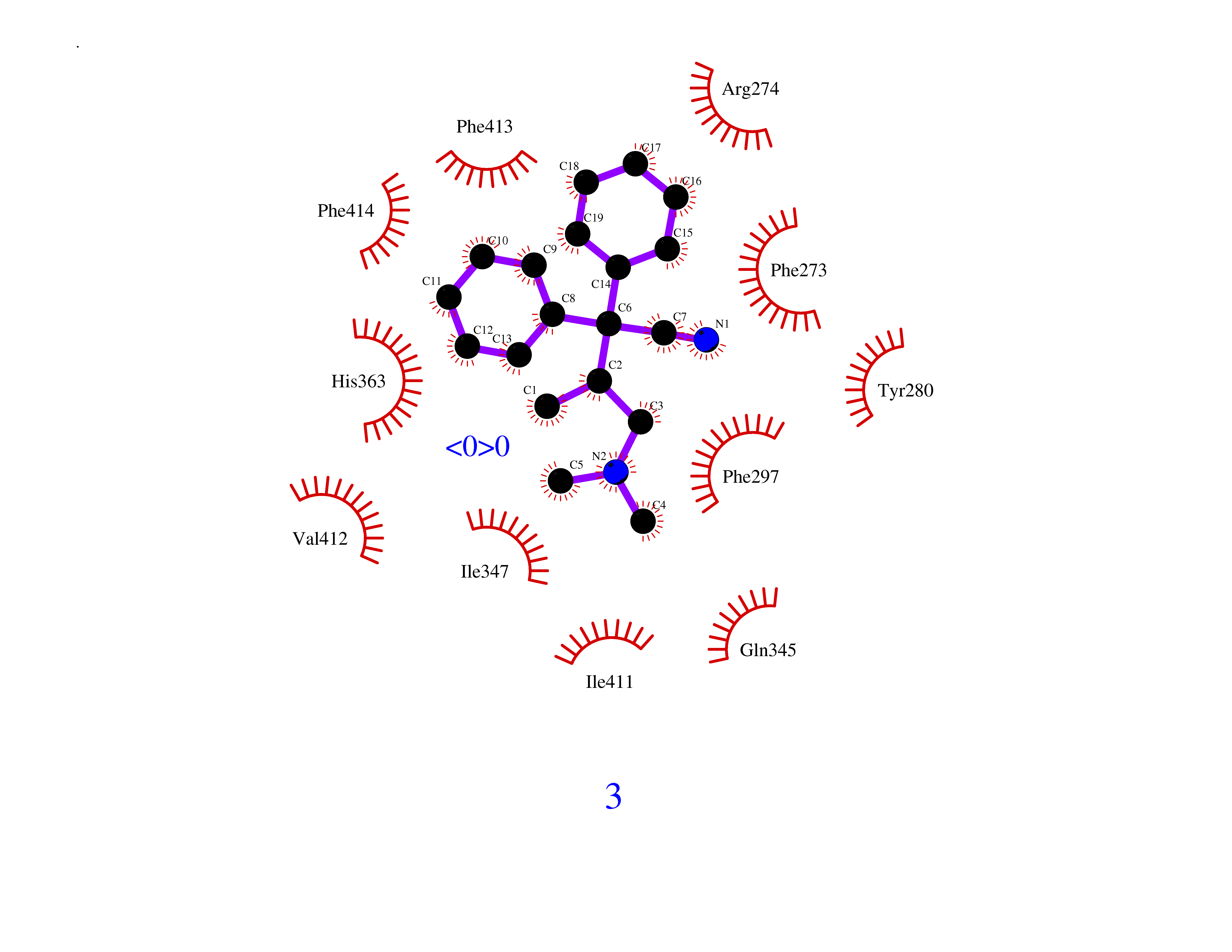

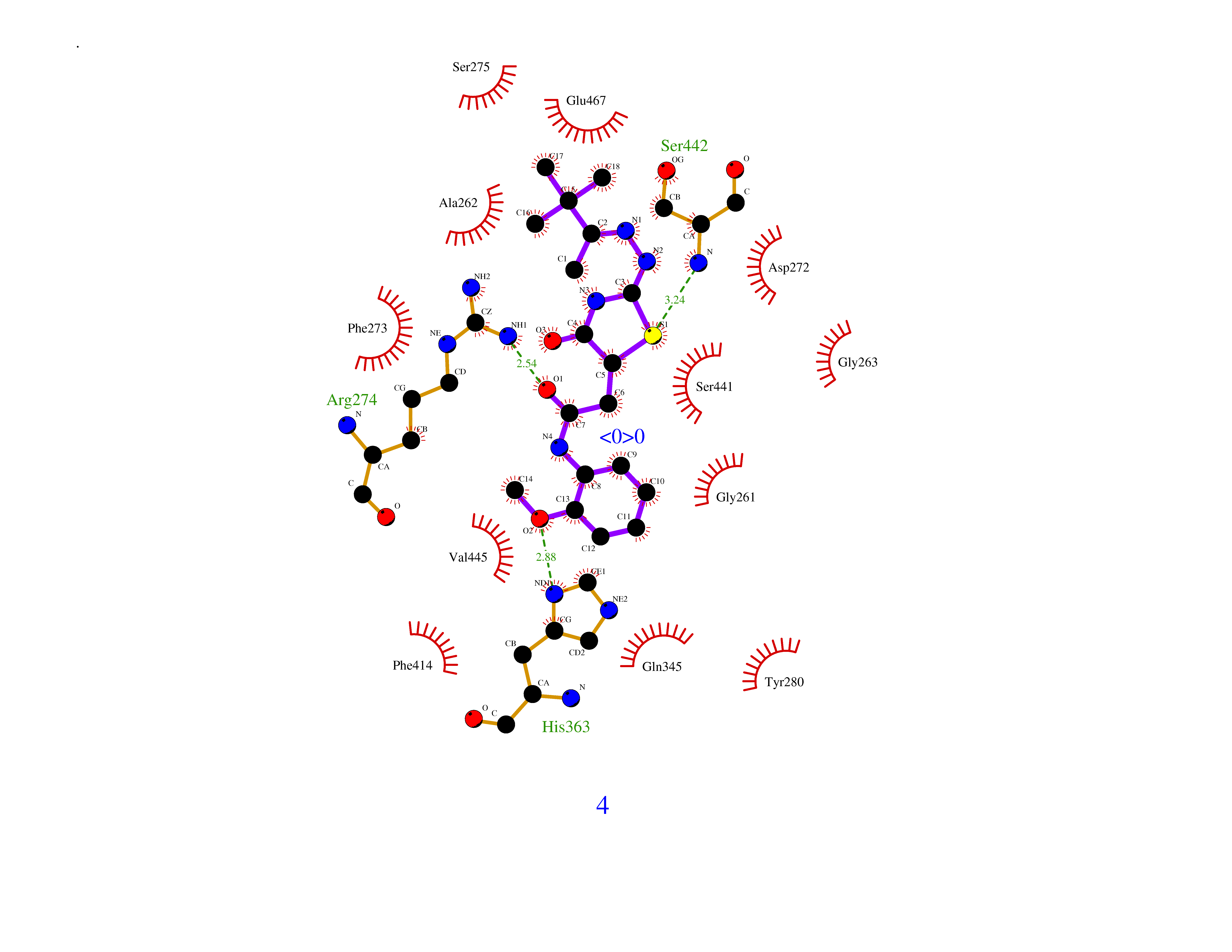

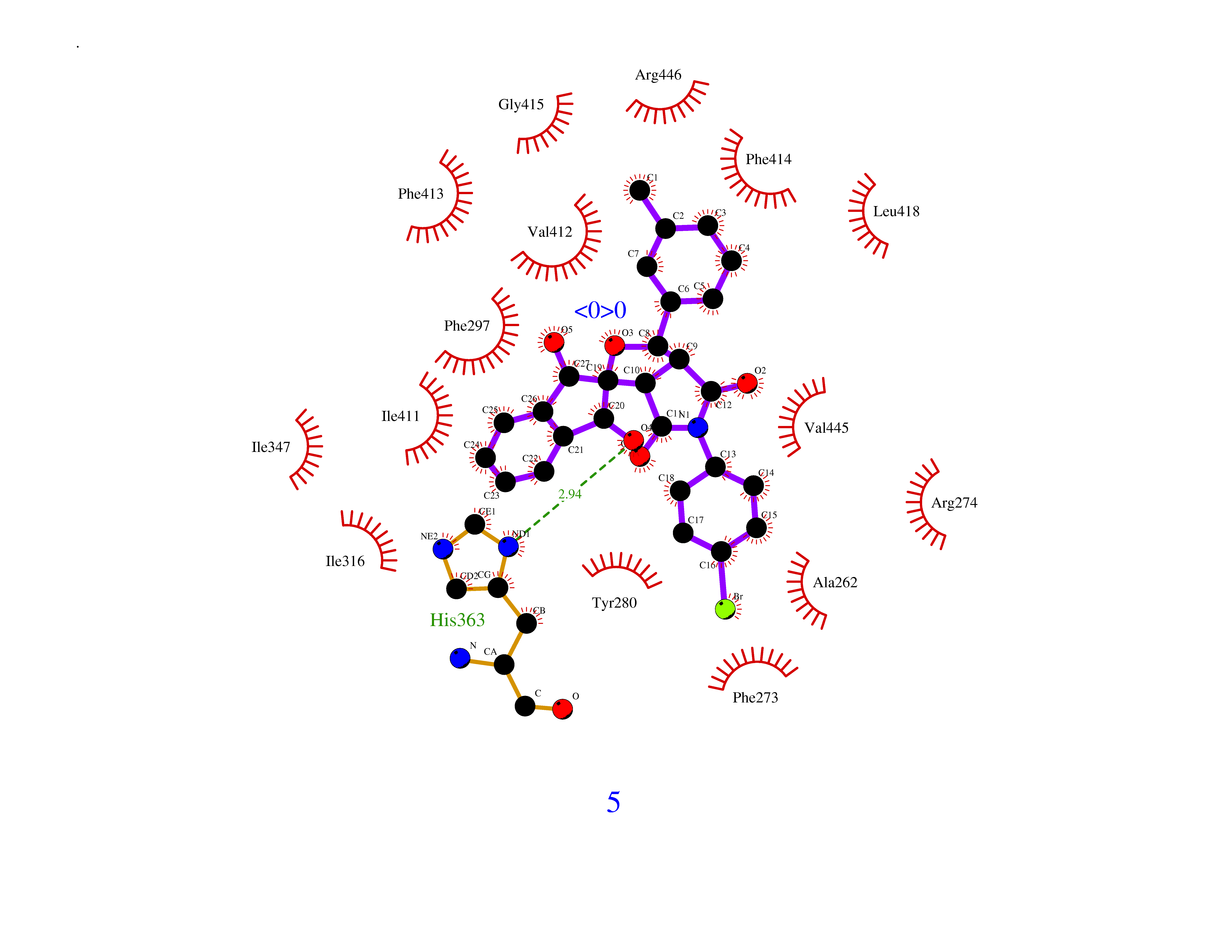

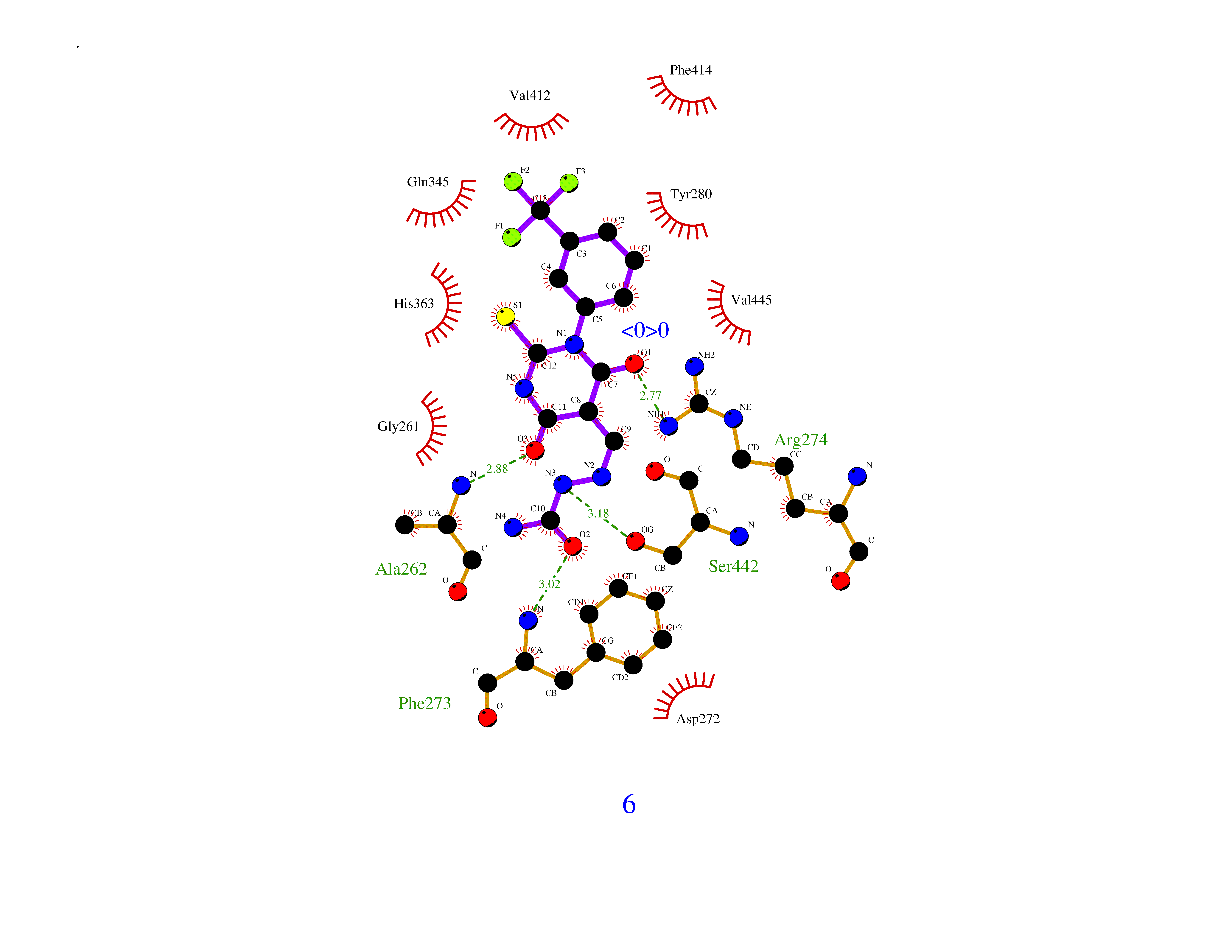

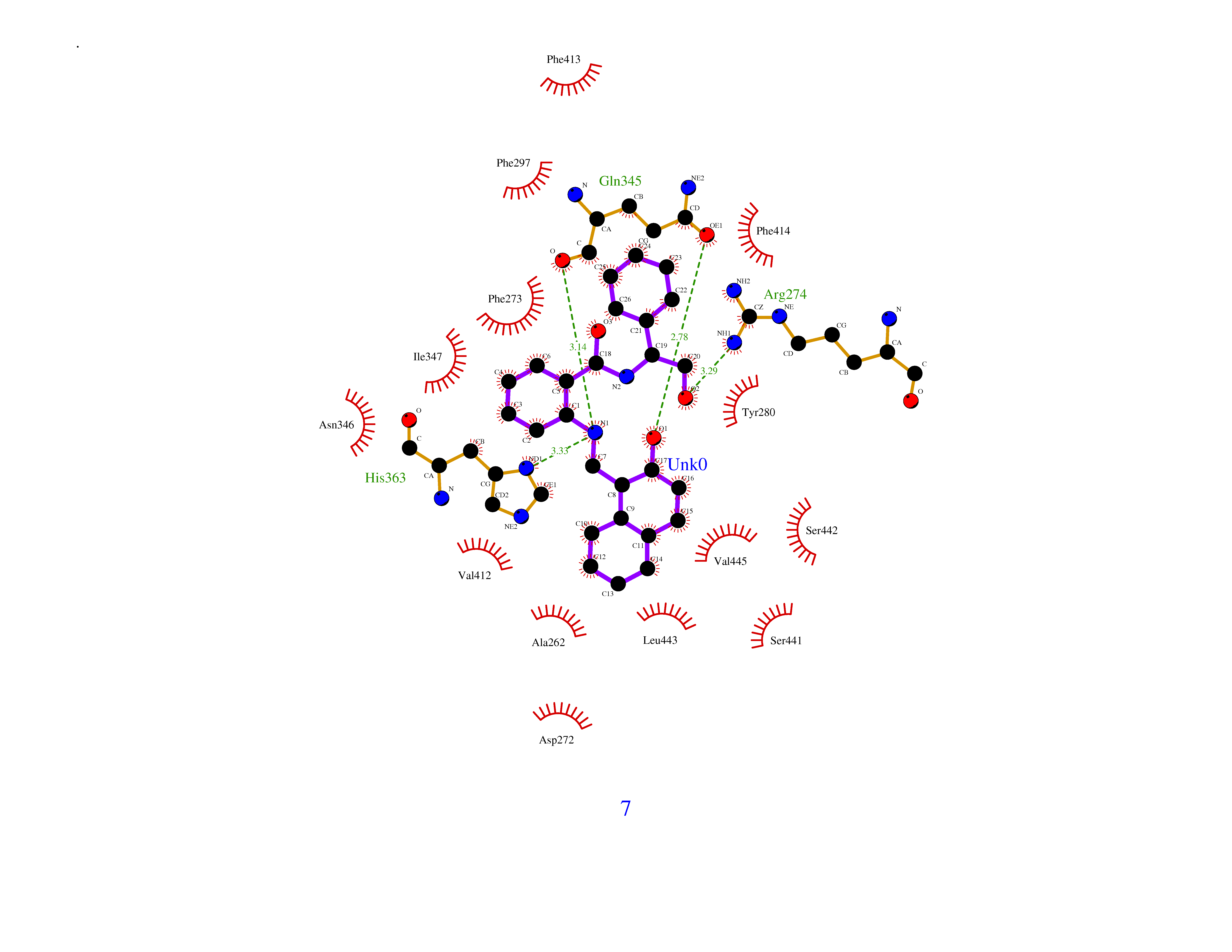

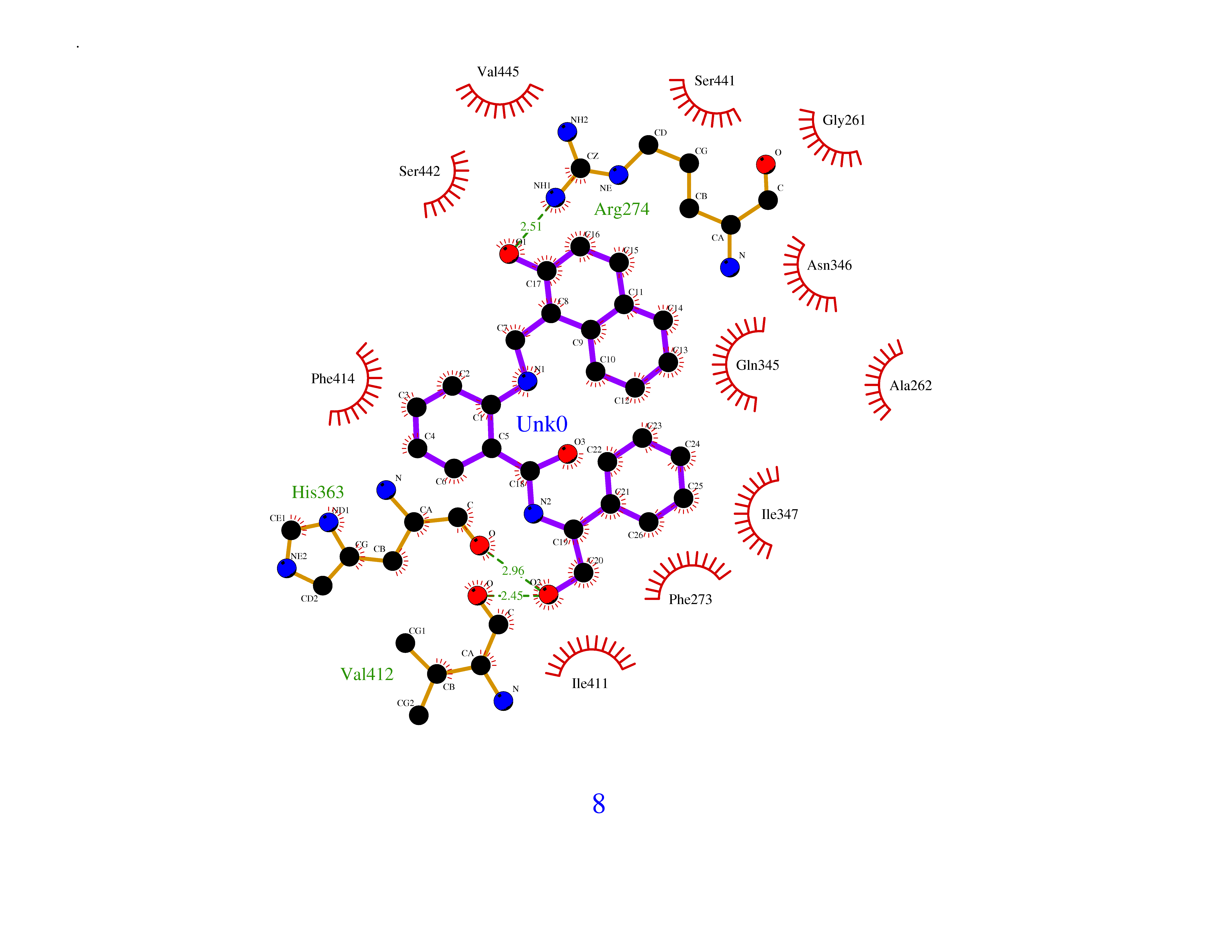

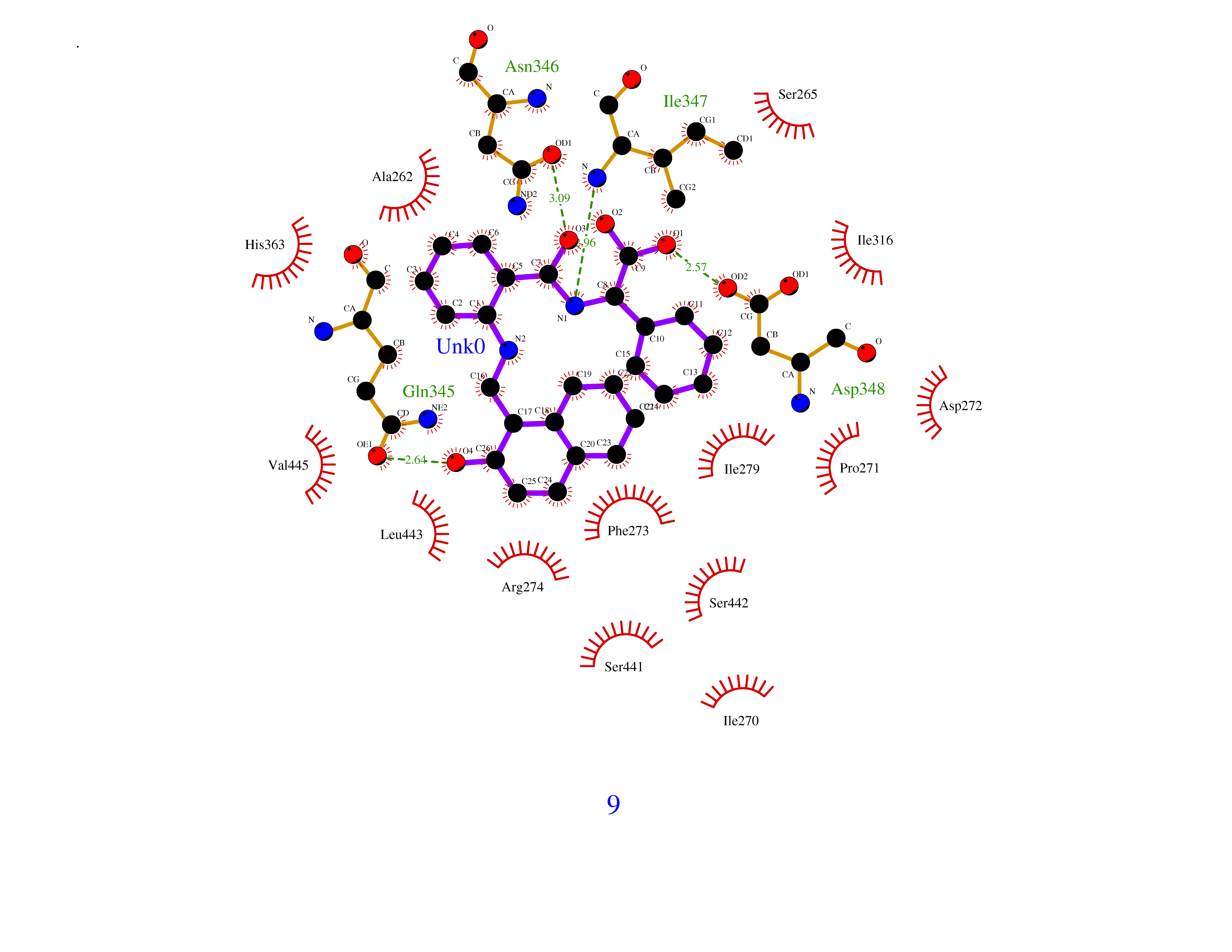

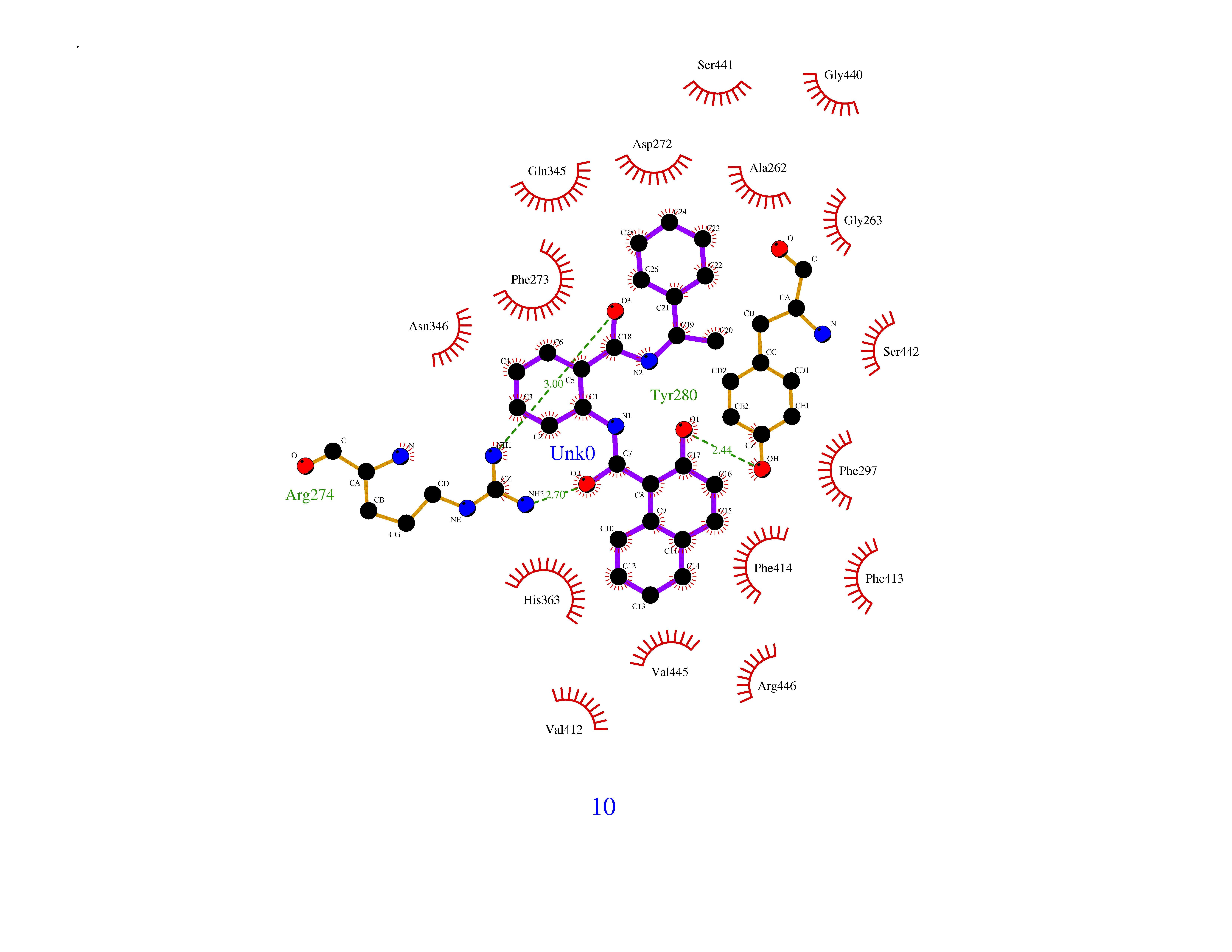


**Figure S3:** 2D plots of all the inhibitors human Sirt1. Here 1represnts Salermide, 2 with sirtinol, 3,4, 5 and 6 with sale1, sale2, sale3, sale4 and sale5 and 7,8,9 and 10 with sirt1,sirt2, sirt3 and sirt4.

**
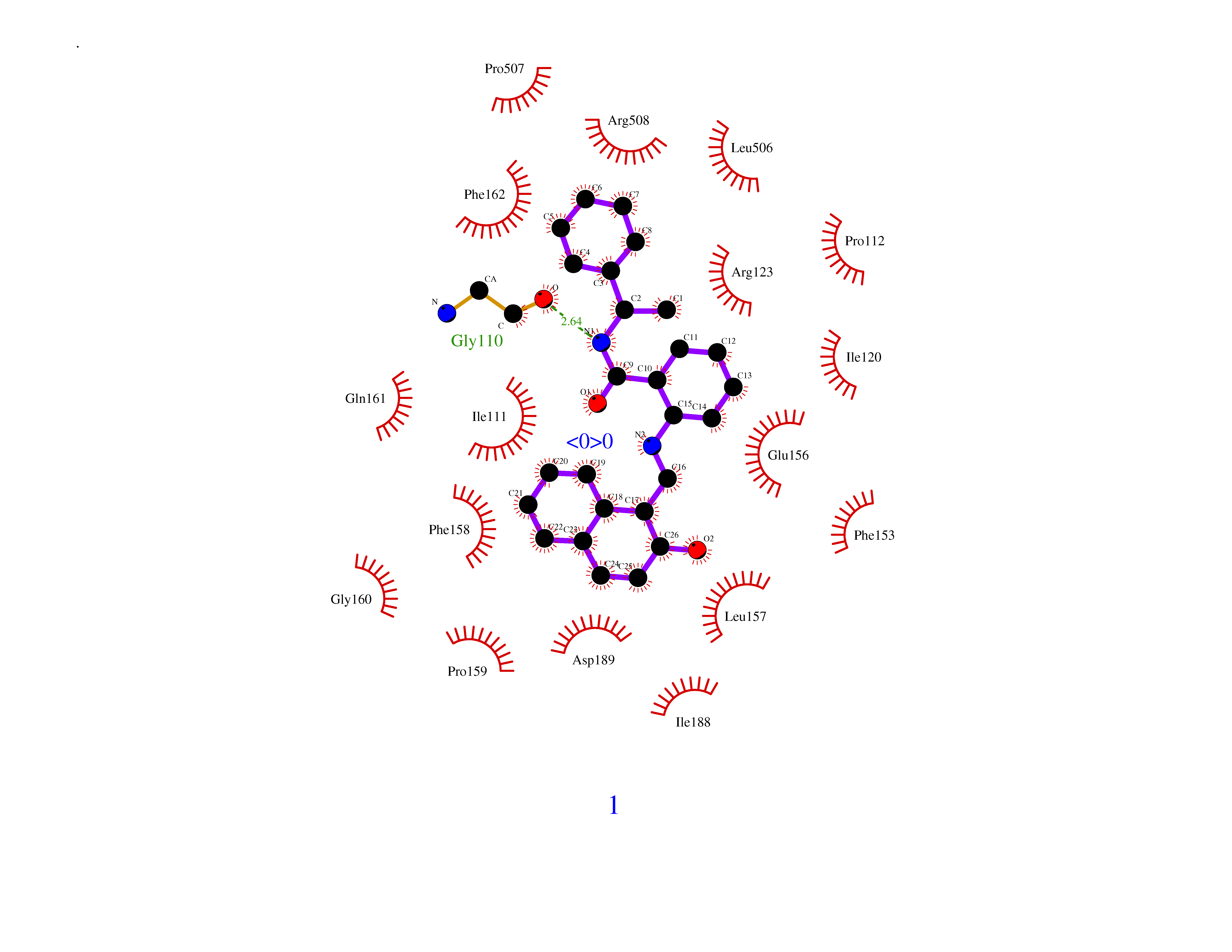

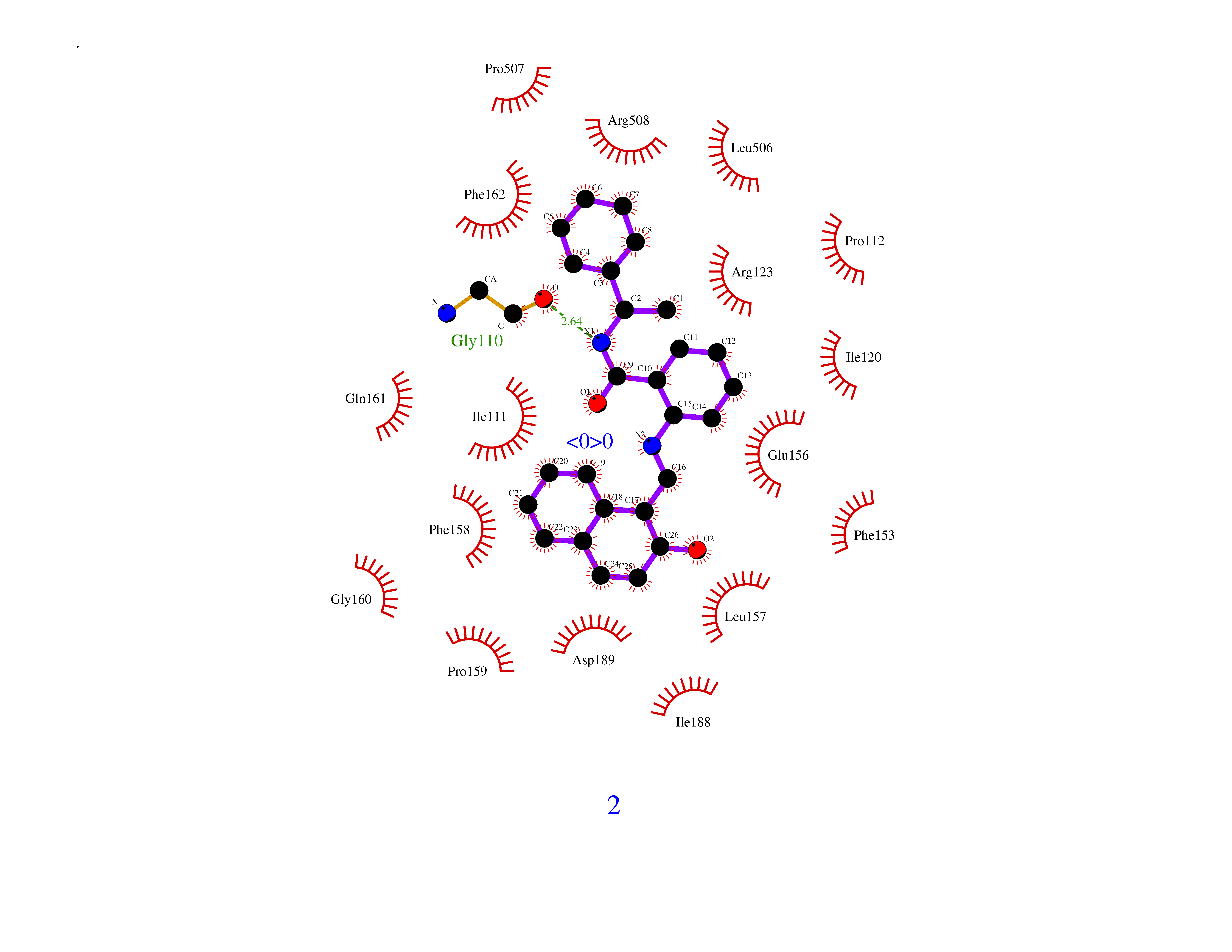

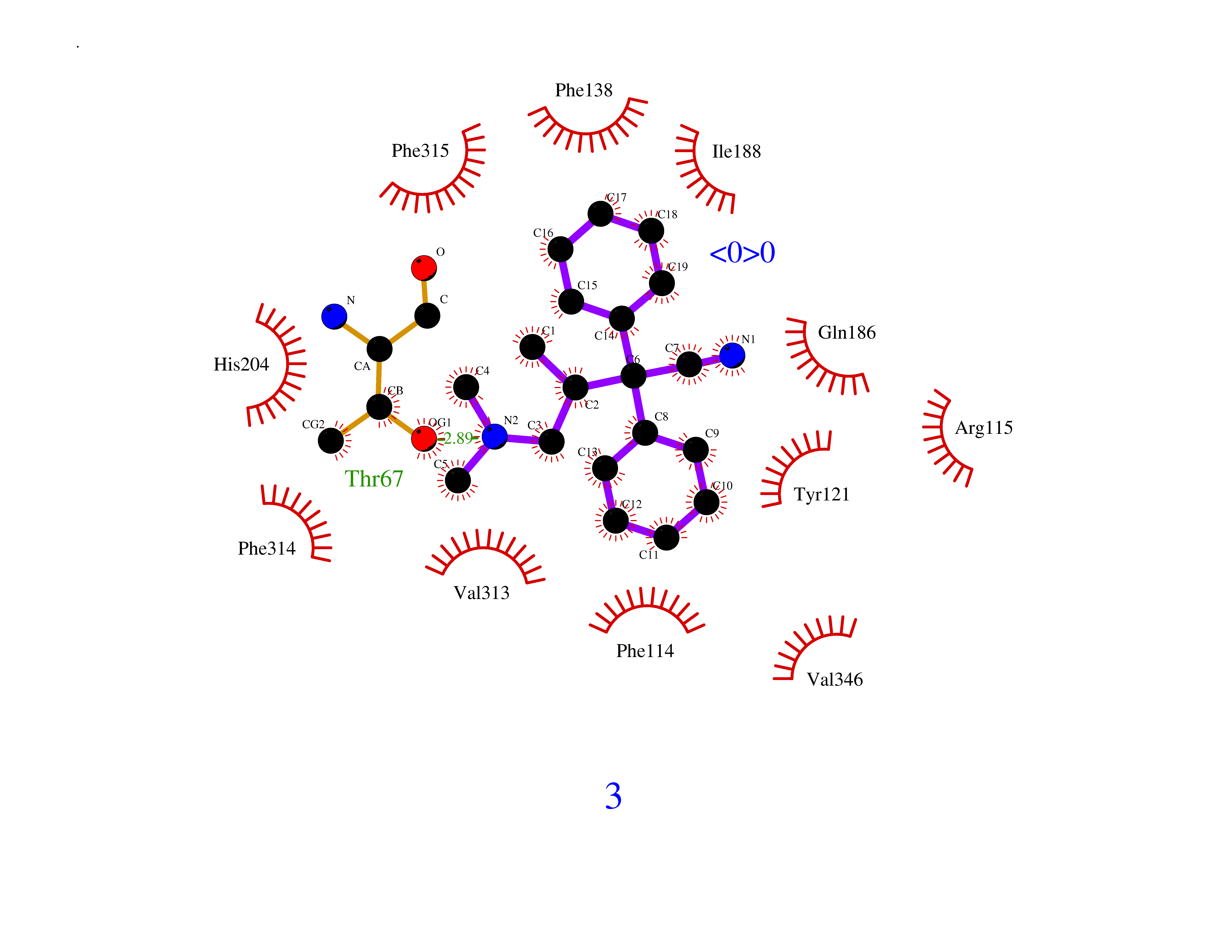

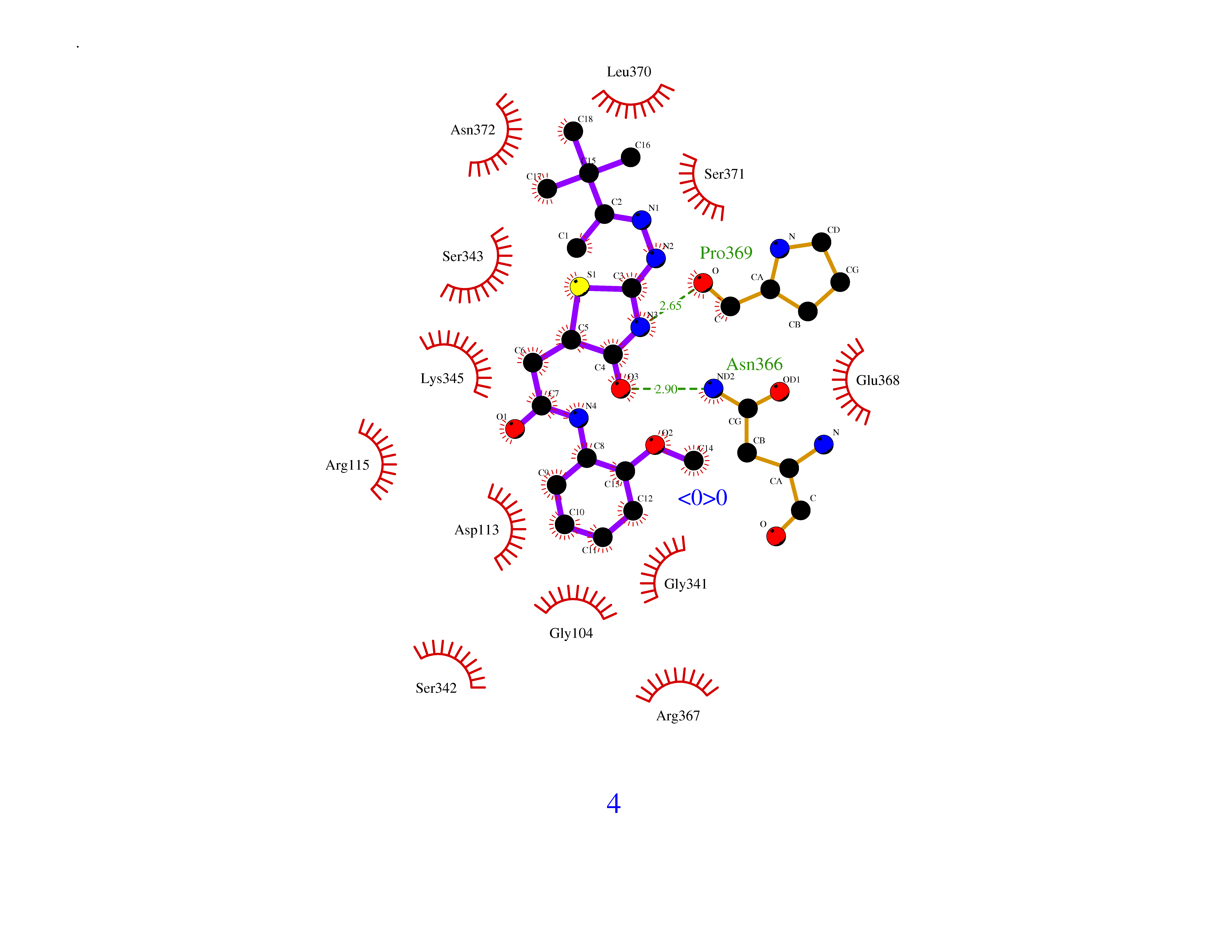

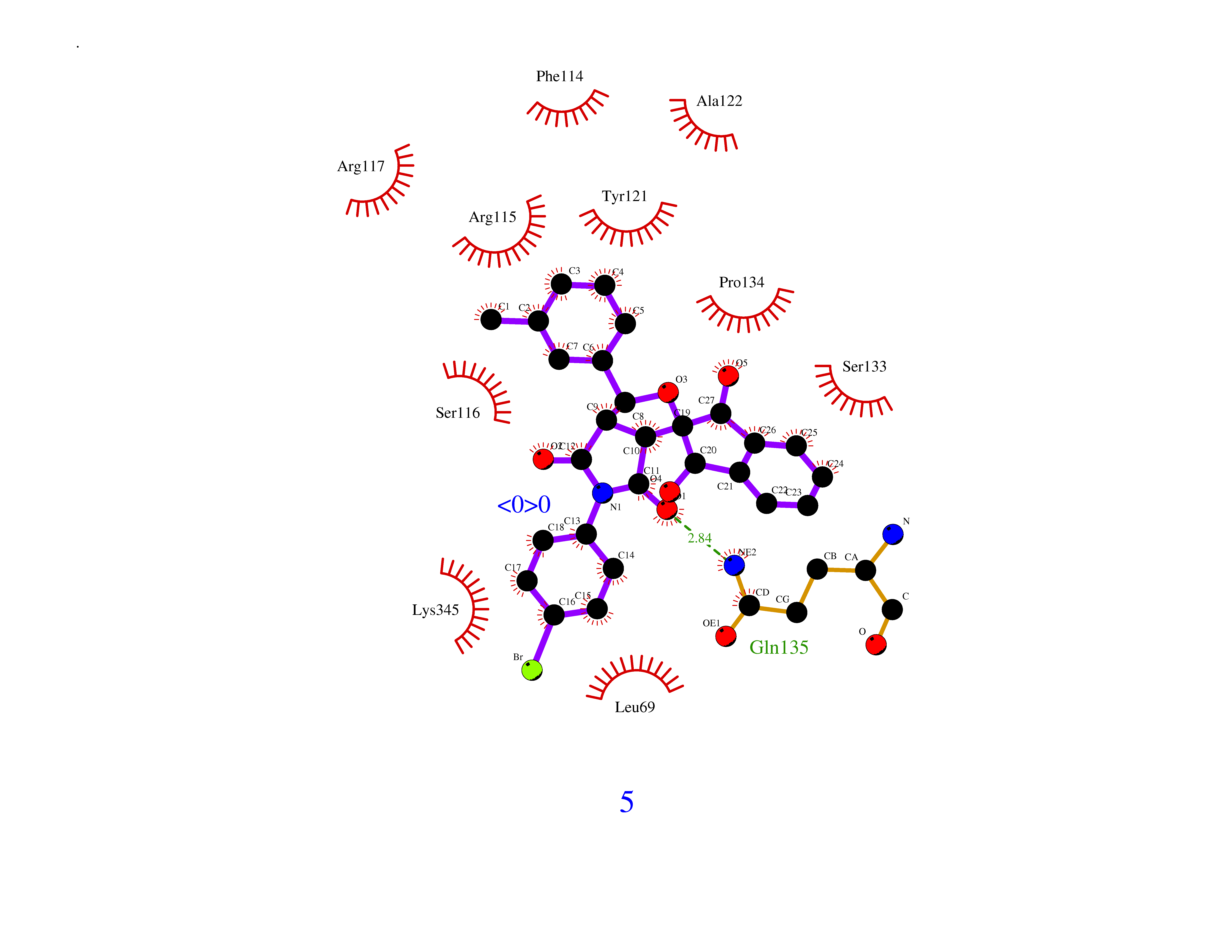

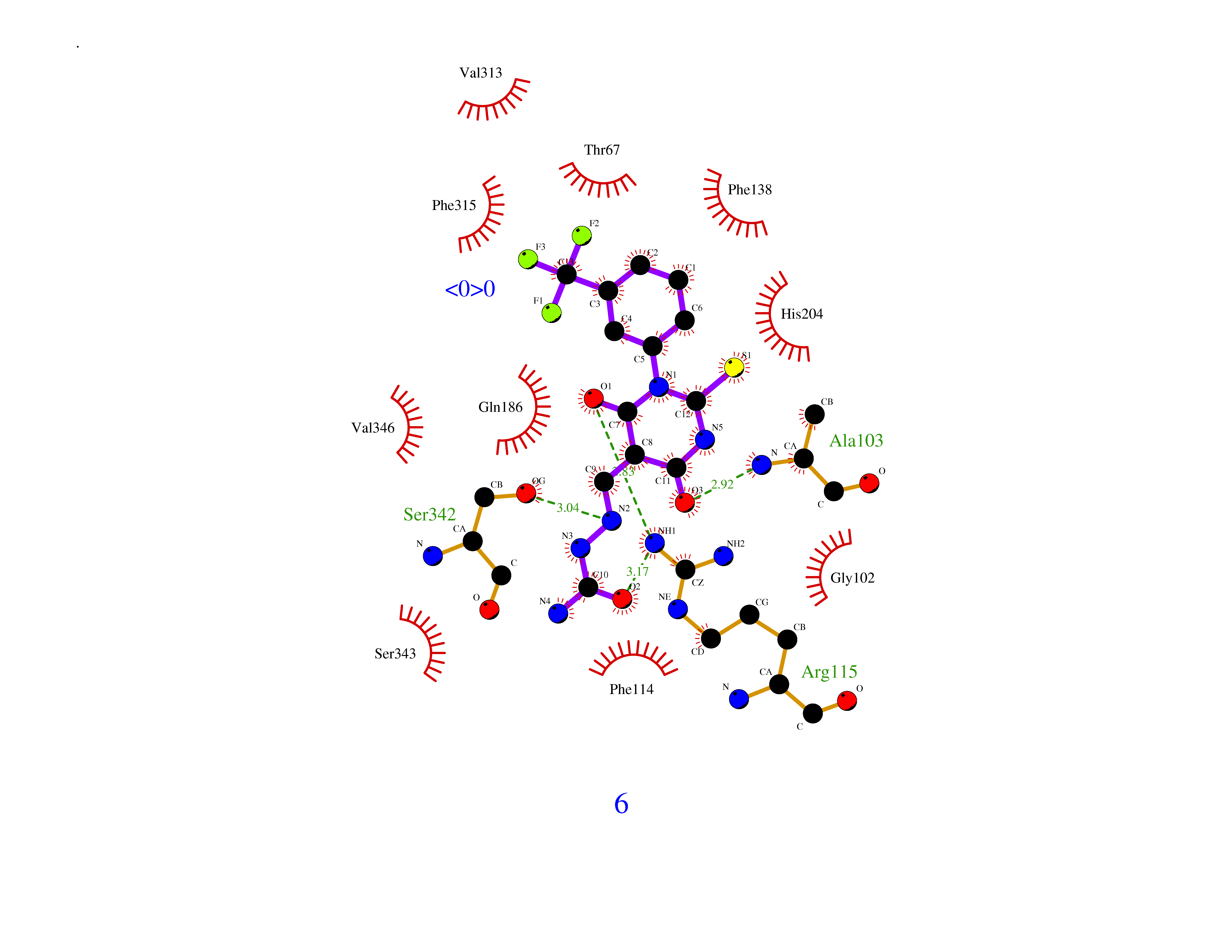

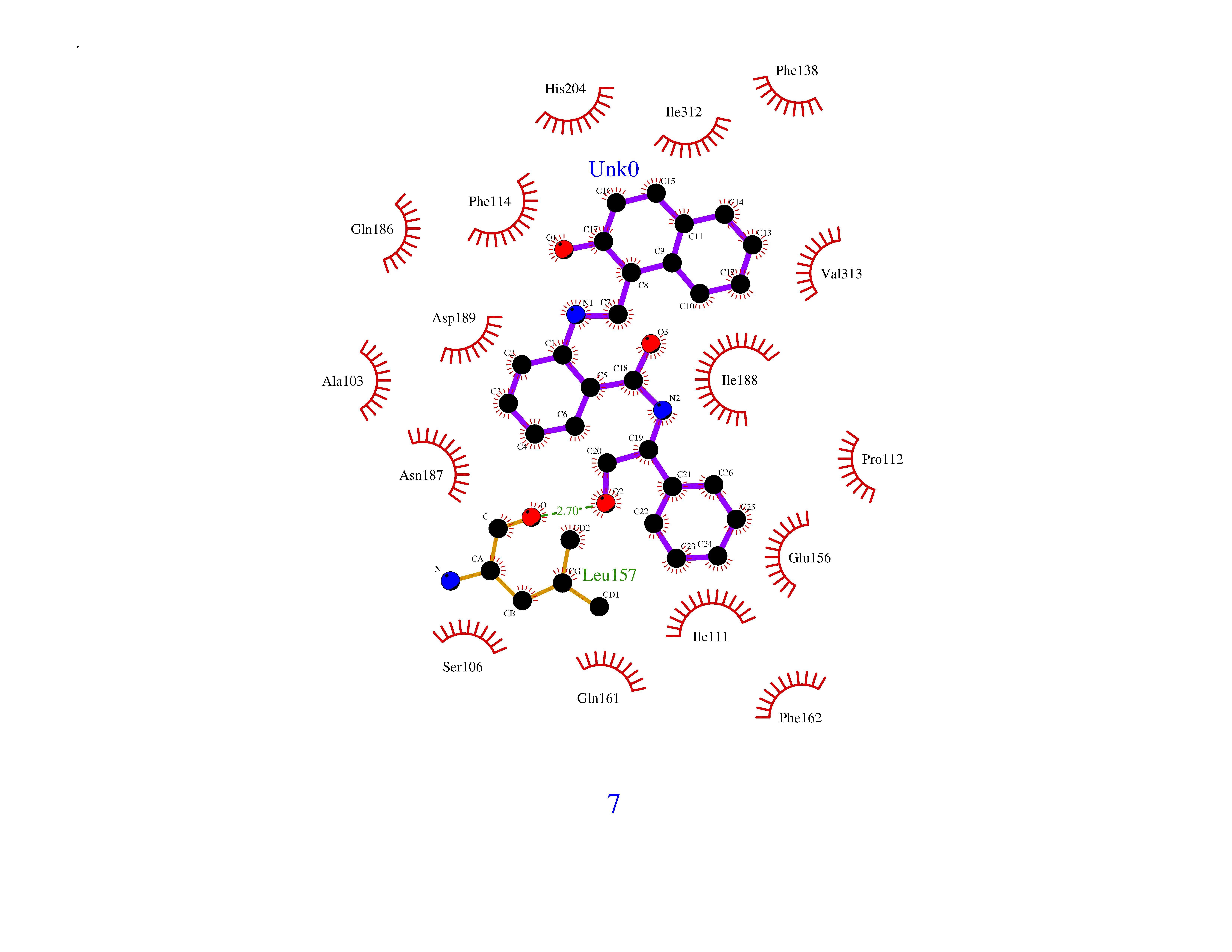

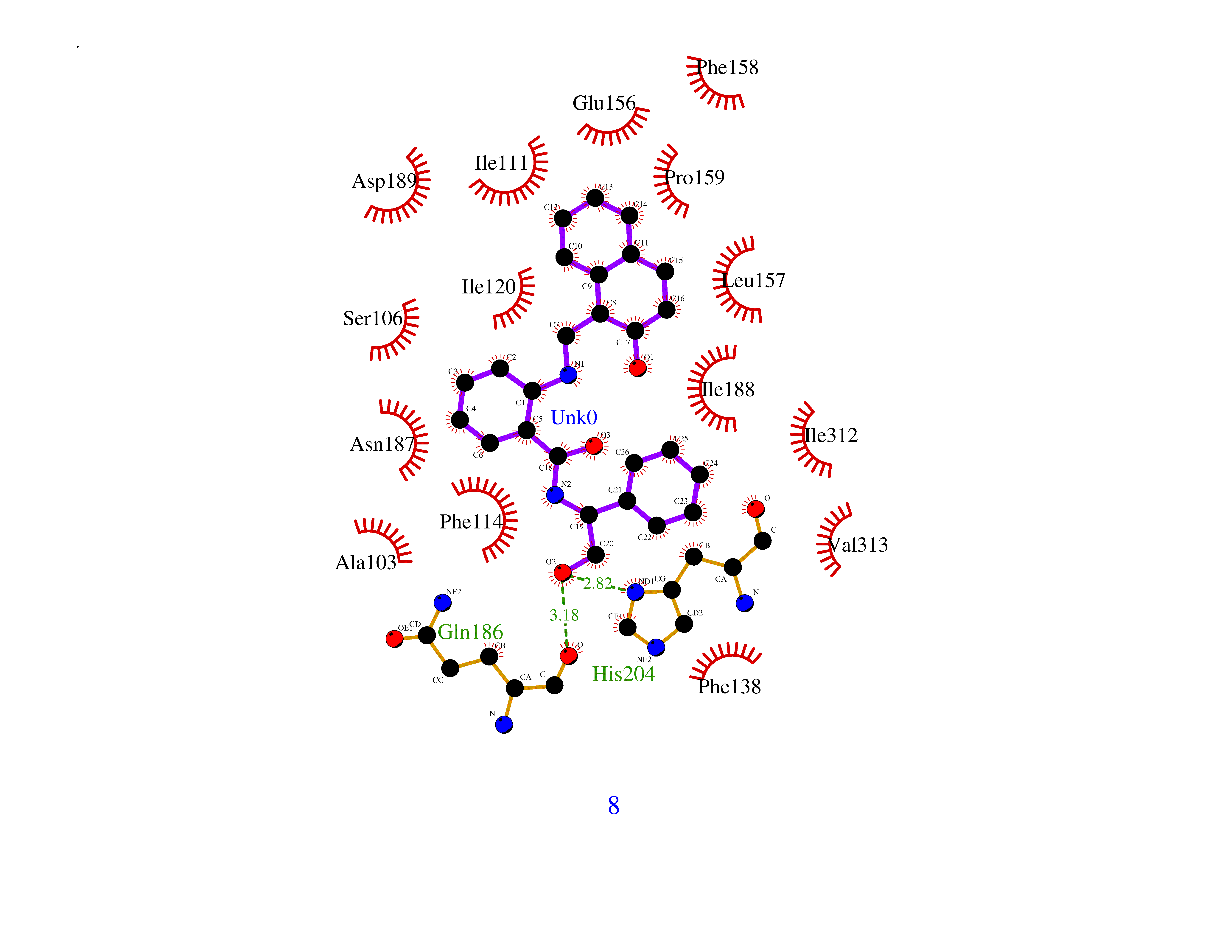

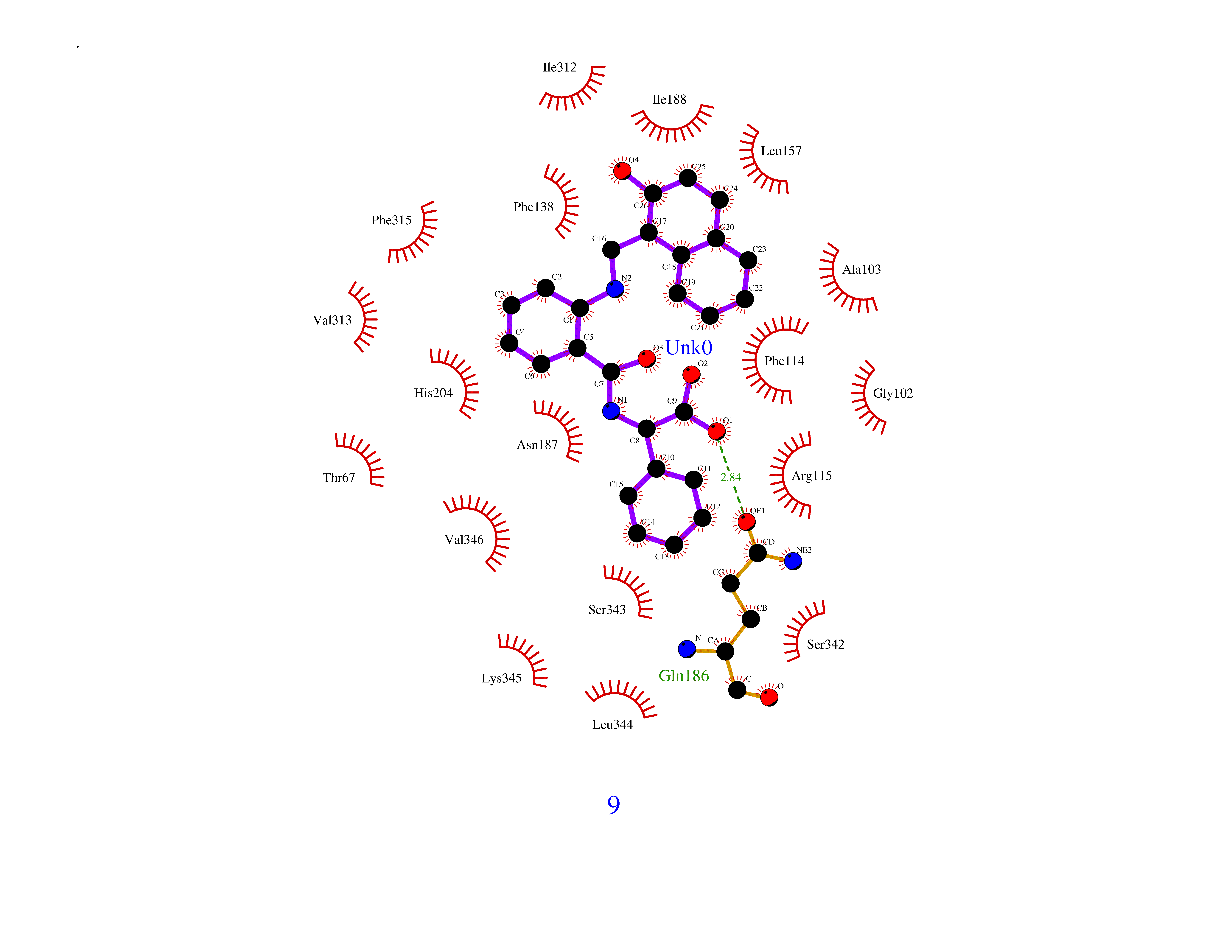

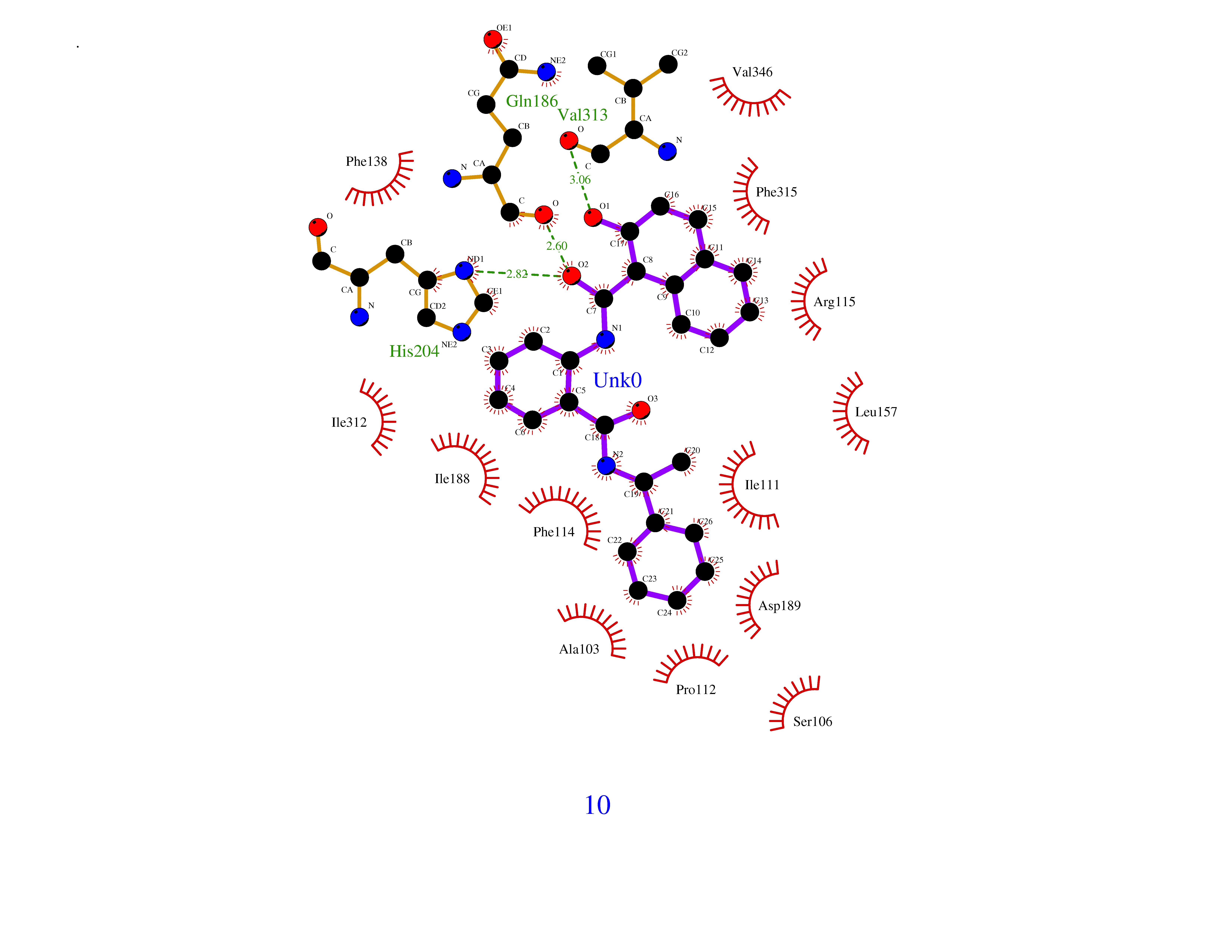
**

**Figure S4:** 2D plots of all the inhibitors with Schistosoma Sirt1. Here 1 represnts Salermide, 2 with sirtinol, 3,4, 5 and 6 with sale1, sale2, sale3, sale4 and sale5 and 7,8,9 and 10 with sirt1,sirt2, sirt3 and sirt4.

**Table S 1: ADMET screening results**

|  | Blood-Brain Barrier | Human Intestinal Absorption | Caco-2 Permeability | P-glycoprotein Substrate | P-glycoprotein Inhibitor | Renal Organic Cation Transporter inhibitor | CYP450 2C9 Substrate | CYP450 2D6 Substrate | CYP450 3A4 Substrate | CYP450 1A2 Inhibitor | CYP450 2C9 Inhibitor | CYP450 2D6 Inhibitor | CYP450 2C19 Inhibitor | CYP450 3A4 Inhibitor | CYP Inhibitory Promiscuity | Human Ether-a-go-go-Related Gene Inhibition | AMES Toxicity | Carcinogens | Fish Toxicity | Tetrahymena Pyriformis Toxicity | Honey Bee Toxicity | Biodegradation | Acute Oral Toxicity |
| --- | --- | --- | --- | --- | --- | --- | --- | --- | --- | --- | --- | --- | --- | --- | --- | --- | --- | --- | --- | --- | --- | --- | --- |
| Salermide | Yes  0.9088 | Yes 0.9873 | Yes 0.6348 | No 0.7079 | No 0.7791 | No 0.8411 | No 0.5924 | No 0.6338 | Yes 0.5263 | Yes 0.9184 | No 0.6492 | No 0.9187 | No 0.6366 | No 0.5590 | Yes 0.7047 | Weak 0.9851 | Yes 0.9352 | No 0.6450 | Yes 0.8887 | Yes 0.9439 | Low 0.7867 | No 0.9880 | Category III  0.4354 |
| Sale1 | Yes 0.9000 | Yes 0.8364 | Yes 0.7074 | No 0.5361 | No 0.8906 | No 0.5991 | No 0.7901 | No 0.7868 | No 0.5000 | No 0.8697 | No 0.8757 | Yes 0.7516 | No 0.8817 | Yes 0.7238 | Low 0.5725 | Waek 0.9089 | Yes  0.8604 | Yes  0.5000 | Yes  0.8069 | Yes  0.9368 | Low 0.6371 | No 0.9897 | Category III 0.6172 |
| Sale2 | No 0.5997 | Yes 0.9387 | No 0.6169 | Yes 0.6586 | No 0.6369 | No 0.8674 | No 0.7869 | No 0.8097 | Yes 0.5832 | No 0.6614 | Yes 0.6037 | No 0.9178 | No 0.5000 | Yes 0.5876 | Yes 0.7490 | Weak 0.9792 | No 0.6308 | No 0.7801 | Yes 0.9739 | Yes 0.9666 | Low0.7145 | No 1.0000 | Category three 0.5781 |
| Sale3* under danger category of carcinogens class III | Yes 0.9740 | Yes 1.0000 | Yes 0.5560 | No 0.7365 | No 0.6214 | No 0.9076 | No 0.7874 | No 0.8567 | Yes 0.6024 | No 0.7908 | No 0.5218 | No 0.9094 | Yes 0.5083 | No 0.7554 | Yes 0.6666 | Weak 0.9963 | No 0.7360 | No 0.7588 | Yes 0.9854 | Yes 0.9884 | Low 0.8394 | No 0.9975 | Category three 0.6033 |
| Sale4 | Yes 0.6363 | Yes 0.7623 | No 0.5841 | No 0.5427 | No 0.5493 | No 0.8834 | No 0.7983 | No 0.8115 | No 0.6092 | No 0.6632 | No 0.5489 | No 0.8557 | No 0.5000 | No 0.8048 | Low 0.5391 | Weak 0.9931 | No 0.5604 | No 0.6473 | Yes 0.9984 | Yes 0.9774 | Low 0.8332 | No 1.0000 | Category III 0.6323 |
| Sirtinol | Yes 0.9088 | Yes 0.9873 | Yes 0.6348 | No 0.7079 | No 0.7655 | No 0.8411 | No 0.5924 | No 0.6338 | Yes 0.5263 | Yes 0.9184 | No 0.6492 | No 0.9187 | No 0.6366 | No 0.5590 | Yes 0.7047 | Weak 0.9851 | Yes 0.9352 | No 0.6450 | Yes 0.8887 | Yes 0.9439 | Low 0.7867 | No 0.9880 | Category III 0.4354 |
| Sirt 1 | No 0.7493 | Yes 0.8094 | No 0.6828 | Yes 0.5382 | No 0.6564 | No 0.7816 | No 0.6922 | No 0.7235 | No 0.5730 | No 0.8101 | No 0.8842 | No 0.8444 | No 0.7245 | No 0.9565 | Low 0.7634 | Weak 0.9067 | No 0.5422 | No 0.9294 | Yes 0.6458 | Low 0.6764 | Low 0.6899 | No 0.7830 | Category III 0.6061 |
| Sirt 2 | No 0.7493 | Yes 0.8094 | No 0.6828 | Yes 0.5382 | No 0.6564 | No 0.7816 | No 0.6922 | No 0.7235 | No 0.5730 | No 0.8101 | No 0.8842 | No 0.8444 | No 0.7245 | No 0.9565 | Low 0.7634 | Weak 0.9067 | No 0.5422 | No 0.9294 | Yes 0.6458 | Low 0.6764 | Low 0.6899 | No 0.7830 | Category III 0.6061 |
| Sirt 3 | No 0.7709 | No 0.5434 | No 0.7391 | No 0.5188 | No 0.8332 | No 0.8511 | No 0.6790 | No 0.7692 | No 0.5000 | No 0.8677 | No 0.9081 | No 0.8779 | No 0.8319 | No 0.9665 | Low 0.8174 | Weak 0.9415 | No 0.6809 | No 0.9572 | Yes 0.7794 | Low 0.6558 | Low 0.6812 | No 0.5611 | Category III 0.6091 |
| Sirt 4 | Yes 0.5966 | Yes 0.9576 | No 0.5654 | Yes 0.5169 | No 0.7759 | No 0.8060 | no 0.6128 | No 0.7648 | Yes 0.5472 | No 0.7951 | No 0.8304 | No 0.9124 | No 0.8116 | No 0.9043 | Low 0.6130 | Weak 0.9681 | No 0.6652 | No 0.9162 | Yes 0.7796 | Yes 0.8244 | Low 0.6957 | No 0.9067 | Category III |
